# Intravenous Thrombolysis in Patients With Ischemic Stroke and Recent Ingestion of Direct Oral Anticoagulants

**DOI:** 10.1001/jamaneurol.2022.4782

**Published:** 2023-01-03

**Authors:** Thomas R. Meinel, Duncan Wilson, Henrik Gensicke, Jan F. Scheitz, Peter Ringleb, Ioana Goganau, Johannes Kaesmacher, Hee-Joon Bae, Do Yeon Kim, Pawel Kermer, Kentaro Suzuki, Kazumi Kimura, Kosmas Macha, Masatoshi Koga, Shinichi Wada, Valerian Altersberger, Alexander Salerno, Logesh Palanikumar, Andrea Zini, Stefano Forlivesi, Lars Kellert, Johannes Wischmann, Espen S. Kristoffersen, James Beharry, P. Alan Barber, Jae Beom Hong, Carlo Cereda, Eckhard Schlemm, Yusuke Yakushiji, Sven Poli, Ronen Leker, Michele Romoli, Marialuisa Zedde, Sami Curtze, Benno Ikenberg, Timo Uphaus, David Giannandrea, Pere Cardona Portela, Roland Veltkamp, Annemarei Ranta, Marcel Arnold, Urs Fischer, Jae-Kwan Cha, Teddy Y. Wu, Jan C. Purrucker, David J. Seiffge, and the DOAC-IVT Writing Group, Georg Kägi, Stefan Engelter, Christian H. Nolte, Bernd Kallmünzer, Patrik Michel, Timothy J. Kleinig, John Fink, Ole Morten Rønning, Bruce Campbell, Paul J. Nederkoorn, Götz Thomalla, Takenobu Kunieda, Khouloud Poli, Yannick Béjot, Yannie Soo, Carlos Garcia-Esperon, Georges Ntaios, Charlotte Cordonnier, João Pedro Marto, Guido Bigliardi, François Lun, Philip M. C. Choi, Thorsten Steiner, Xavier Ustrell, David Werring, Susanne Wegener, Alessandro Pezzini, Houwei Du, Joan Martí-Fàbregas, David Cánovas-Vergé, Daniel Strbian, Visnja Padjen, Shadi Yaghi, Christoph Stretz, Joon-Tae Kim

**Affiliations:** 1 Stroke Research Center Bern, Department of Neurology, Inselspital, Bern University Hospital, University of Bern, Switzerland; 2 Department of Neurology, Christchurch Hospital, Christchurch, New Zealand; 3 New Zealand Brain Research Institute, Christchurch, New Zealand; 4 Stroke Center, Department of Neurology, University Hospital Basel, Basel, Switzerland; 5 Neurology and Neurorehabilitation, University Department of Geriatric Medicine Felix Platter, University of Basel, Basel, Switzerland; 6 Department of Neurology, Berlin Institute of Health, Charité–Universitätsmedizin Berlin, Berlin, Germany; 7 German Center for Cardiovascular Research Partner Site Berlin, Germany; 8 Center for Stroke Research Berlin, Charité–Universitätsmedizin Berlin, Berlin, Germany; 9 Department of Neurology, Heidelberg University Hospital, Heidelberg, Germany; 10 Institute of Diagnostic and Interventional Neuroradiology, Stroke Research Center Bern, Inselspital, Bern University Hospital, University of Bern, Switzerland; 11 Department of Neurology, Cerebrovascular Disease Center, Seoul National University Bundang Hospital, Seoul National University College of Medicine, Seongnam-si, South Korea; 12 Department of Neurology, Friesland Kliniken, Sande, Germany; 13 Department of Neurology, University Medicine Göttingen, Göttingen, Germany; 14 Department of Neurology, Nippon Medical School, Tokyo, Japan; 15 Department of Neurology, Universitätsklinikum Erlangen, Friedrich-Alexander-Universität Erlangen-Nürnberg, Erlangen, Germany; 16 Department of Cerebrovascular Medicine, National Cerebral and Cardiovascular Center, Osaka, Japan; 17 Stroke Center, Neurology Service, Department of Clinical Neurosciences, Lausanne University Hospital and University of Lausanne, Lausanne, Switzerland; 18 Department of Neurology, Royal Adelaide Hospital, Adelaide, Australia; 19 IRCCS Istituto Delle Scienze Neurologiche Di Bologna, Department of Neurology and Stroke Center, Maggiore Hospital, Bologna, Italy; 20 Department of Neurology, University Hospital, LMU Munich, Munich, Germany; 21 Department of Neurology, Akershus University Hospital, Lørenskog, Norway; 22 Department of General Practice, Institute of Health and Society (HELSAM), University of Oslo, Oslo, Norway; 23 Department of Medicine and Neurology, Melbourne Brain Centre at The Royal Melbourne Hospital, University of Melbourne, Parkville, Australia; 24 Department of Medicine, Auckland University, Auckland, New Zealand; 25 Stroke Center and Department of Neurology, Neurocenter of Southern Switzerland, Lugano, Switzerland; 26 Klinik und Poliklinik Für Neurologie, Kopf, und Neurozentrum, Universitätsklinikum Hamburg-Eppendorf, Hamburg, Germany; 27 Department of Neurology Kansai Medical University, Hirakata, Japan; 28 Department of Neurology and Stroke, University of Tübingen, Tübingen, Germany; 29 Hertie-Institute for Clinical Brain Research, University of Tübingen, Tübingen, Germany; 30 Department of Neurology, Hadassah-Hebrew University Medical Center, Jerusalem, Israel; 31 Neurology and Stroke Unit, Department of Neuroscience, Bufalini Hospital, Cesena, Italy; 32 Neurology Unit, Stroke Unit, Azienda Unità Sanitaria Locale-IRCCS di Reggio Emilia, Reggio Emilia, Italy; 33 Neurology, University of Helsinki and Helsinki University Hospital, Helsinki, Finland; 34 Department of Neurology, School of Medicine, Klinikum rechts der Isar, Technical University of Munich, Munich, Germany; 35 Department of Neurology, University Medical Center of the Johannes Gutenberg University Mainz, Germany; 36 Division of Neurology and Stroke Unit, Department of Neurology, Gubbio and Città di Castello Hospital, Perugia, Italy; 37 Department of Neurology, Stroke Unit, Hospital Universitari Bellvitge, Barcelona, Spain; 38 Klinik für Neurologie, Alfried Krupp Krankenhaus, Essen, Germany; 39 Department of Brain Sciences, Imperial College London, London, United Kingdom; 40 Department of Medicine, University of Otago, Wellington, New Zealand; 41 Department of Neurology, Capital and Coast District Health Board, Wellington, New Zealand; 42 Department of Neurology, Dong-A University Hospital, Busan, South Korea; 43 Department of Neurology, Cantonal Hospital, St Gallen, Switzerland; 44 Berlin Institute of Health at Charité–Universitätsmedizin Berlin, Berlin, Germany; 45 German Center for Cardiovascular Research Partner Site Berlin, Berlin, Germany; 46 German Center for Neurodegenerative Diseases Partner Site Berlin, Berlin, Germany; 47 Department of Neurology, Canterbury District Health Board, Christchurch, New Zealand; 48 Department of Neurology, Amsterdam UMC, Location AMC, University of Amsterdam, Amsterdam, the Netherlands; 49 Department of Neurology, University Hospital Dijon, Dijon, France; 50 Division of Neurology, Department of Medicine and Therapeutics Faculty of Medicine, The Chinese University of Hong Kong, Hong Kong Special Administrative Region, China; 51 John Hunter Hospital, Newcastle, Australia; 52 Hunter Medical Research Institute, University of Newcastle, Newcastle, Australia; 53 Department of Internal Medicine, Faculty of Medicine, School of Health Sciences, University of Thessaly, Larissa, Greece; 54 Lille Neuroscience and Cognition Research Center, INSERM, CHU Lille, Lille, France; 55 Department of Neurology, Hospital de Egas Moniz, Centro Hospitalar Lisboa Ocidental, Lisbon, Portugal; 56 Department of Neurology, University Hospital Modena, Modena, Italy; 57 Department of Neurology, GHU Paris Psychiatrie et Neurosciences, Université de Paris, Paris, France; 58 Department of Neuroscience, Eastern Health, Eastern Health Clinical School, Monash University, Melbourne, Australia; 59 Department of Neurology, Klinikum Frankfurt Höchst, Frankfurt am Main, Germany; 60 Department of Neurology, Universität Heidelberg, Heidelberg, Germany; 61 Stroke Unit, Neurology Department, Hospital Universitari Joan XXIII, Tarragona, Spain; 62 Stroke Research Centre, UCL Queen Square Institute of Neurology, University College London, London, United Kingdom; 63 Department of Neurology, University Hospital Zurich, University of Zurich, Zurich, Switzerland; 64 Department of Clinical and Experimental Sciences, Neurology Clinic, University of Brescia, Neurology Unit, ASST Spedali Civili, Brescia, Italy; 65 Department of Neurology, Fujian Medical University Union Hospital, Fuzhou, China; 66 Institute of Clinical Neurology, Fujian Medical University, Fuzhou, China; 67 Stroke Unit, Department of Neurology, Institute of Biomedical Research Sant Pau, Hospital de la Santa Creu i Sant Pau, Barcelona, Spain; 68 Neurology Department, Hospital Parc Tauli, Sabadell, Spain; 69 Department of Neurology, Helsinki University Hospital, University of Helsinki, Helsinki, Finland; 70 Neurology Clinic, Clinical Centre of Serbia, Faculty of Medicine, University of Belgrade, Belgrade, Serbia; 71 Department of Neurology, The Warren Alpert Medical School of Brown University, Providence, Rhode Island; 72 Department of Neurology, Chonnam National University Hospital, Gwangju, South Korea

## Abstract

**Importance:**

International guidelines recommend avoiding intravenous thrombolysis (IVT) in patients with ischemic stroke who have a recent intake of a direct oral anticoagulant (DOAC).

**Objective:**

To determine the risk of symptomatic intracranial hemorrhage (sICH) associated with use of IVT in patients with recent DOAC ingestion.

**Design, Setting, and Participants:**

This international, multicenter, retrospective cohort study included 64 primary and comprehensive stroke centers across Europe, Asia, Australia, and New Zealand. Consecutive adult patients with ischemic stroke who received IVT (both with and without thrombectomy) were included. Patients whose last known DOAC ingestion was more than 48 hours before stroke onset were excluded. A total of 832 patients with recent DOAC use were compared with 32 375 controls without recent DOAC use. Data were collected from January 2008 to December 2021.

**Exposures:**

Prior DOAC therapy (confirmed last ingestion within 48 hours prior to IVT) compared with no prior oral anticoagulation.

**Main Outcomes and Measures:**

The main outcome was sICH within 36 hours after IVT, defined as worsening of at least 4 points on the National Institutes of Health Stroke Scale and attributed to radiologically evident intracranial hemorrhage. Outcomes were compared according to different selection strategies (DOAC-level measurements, DOAC reversal treatment, IVT with neither DOAC-level measurement nor idarucizumab). The association of sICH with DOAC plasma levels and very recent ingestions was explored in sensitivity analyses.

**Results:**

Of 33 207 included patients, 14 458 (43.5%) were female, and the median (IQR) age was 73 (62-80) years. The median (IQR) National Institutes of Health Stroke Scale score was 9 (5-16). Of the 832 patients taking DOAC, 252 (30.3%) received DOAC reversal before IVT (all idarucizumab), 225 (27.0%) had DOAC-level measurements, and 355 (42.7%) received IVT without measuring DOAC plasma levels or reversal treatment. The unadjusted rate of sICH was 2.5% (95% CI, 1.6-3.8) in patients taking DOACs compared with 4.1% (95% CI, 3.9-4.4) in control patients using no anticoagulants. Recent DOAC ingestion was associated with lower odds of sICH after IVT compared with no anticoagulation (adjusted odds ratio, 0.57; 95% CI, 0.36-0.92). This finding was consistent among the different selection strategies and in sensitivity analyses of patients with detectable plasma levels or very recent ingestion.

**Conclusions and Relevance:**

In this study, there was insufficient evidence of excess harm associated with off-label IVT in selected patients after ischemic stroke with recent DOAC ingestion.

## Key Points

**Question:**

Is the recent use of direct oral anticoagulants (confirmed ingestion within 48 hours) associated with increased risk of symptomatic intracranial hemorrhage following intravenous thrombolysis for ischemic stroke?

**Findings:**

In this cohort study including 33 207 patients with ischemic stroke who received intravenous thrombolysis at 64 centers in Europe, Asia, Australia, and New Zealand, the risk of symptomatic intracranial hemorrhage was lower among the 832 patients taking direct oral anticoagulant treatment compared with controls with no anticoagulation. This result was consistent among subgroups and different selection strategies.

**Meaning:**

This study found insufficient evidence of excess harm associated with the use of off-label intravenous thrombolysis in selected patients who had taken a direct oral anticoagulant within the previous 48 hours.

## Teaser

This cohort study evaluates the risk of symptomatic intracranial hemorrhage associated with use of intravenous thrombolysis in patients with recent direct oral anticoagulant ingestion.

## Introduction

Use of direct oral anticoagulants (DOACs), including apixaban, dabigatran, edoxaban, and rivaroxaban, has emerged as the primary stroke prevention option in patients with nonvalvular atrial fibrillation (AF).^1^ Additionally, new indications for DOACs are constantly being identified, such as for the treatment of vascular diseases (ie, venous thrombosis, pulmonary embolism, stable coronary heart disease, and peripheral artery disease). It is estimated that since the transition from vitamin K antagonists (VKA) to DOACs, every sixth patient with stroke otherwise qualifying for intravenous thrombolysis (IVT) has a prescription for DOACs.^2,3^ As for VKA with an international normalized ratio (INR) greater than 1.7,^4,5,6^ guidelines recommend the exclusion of patients with recent ingestion of DOACs (within 48 hours previously) from receiving IVT should they have an ischemic stroke.^5,6^ This recommendation is based on the presumption of an increased risk of symptomatic intracranial hemorrhage (sICH), but data to support or refute this presumption are lacking. Data from studies on VKA cannot be translated to DOACs,^7,8,9,10,11,12,13,14^ which have a 50% lower risk of intracranial hemorrhage compared with warfarin.^1^ Furthermore, in experimental ischemic stroke, DOACs did not increase the risk of hemorrhage after IVT, whereas warfarin did.^15,16,17,18^ Different selection strategies, including the use of DOAC reversal agents prior to IVT,^8,9^ selection of patients with low anticoagulant activity at DOAC plasma level measurement,^19^ or point-of-care coagulation assays, have been suggested,^20,21,22^ yet the available evidence originates from comparably small case series^7,8,9,10,11,12,13,14,23^ with single-center experience^19,24,25,26^ and measuring DOAC plasma levels is not possible in many hospitals worldwide,^3^ questioning the feasibility of this approach.

We established an international, multicenter, retrospective cohort study including both published and unpublished data to address this unanswered clinical question. We aimed to compare (1) the risk of sICH in patients with recent ingestion of DOACs receiving IVT as treatment for acute ischemic stroke with that in controls not taking DOACs and (2) the relative safety of different selection strategies for IVT in patients with recent ingestion of DOACs, namely DOAC-level measurements, DOAC reversal prior to IVT, and patients knowingly thrombolyzed but without measurement of DOAC plasma levels or reversal as well as inadvertent IVT with DOAC use subsequently discovered. Our hypothesis was that with currently used selection strategies, the risk of sICH is not higher in patients with recent ingestion of DOACs than in controls who do not take anticoagulants.

## Methods

### Study Design and Data Source

In this investigator-initiated international, multicenter, retrospective analysis, we pooled individual patient data from published and unpublished cohort studies following the Strengthening the Reporting of Observational Studies in Epidemiology (STROBE) reporting guideline. We identified potential contributing centers by a systematic search of the literature based on published studies on IVT in patients with recent ingestion of DOACs or prospective IVT or all-stroke registries (that may include patients with recent ingestion of DOACs). To do so, we searched PubMed/EMBASE using a predefined search strategy combining Medical Subject Headings terms and keywords with the concepts of ischemic stroke, DOAC and IVT (eTable 1 in Supplement 1). We used additional references from articles if relevant information was provided. We included publications in English up to July 31, 2021. The identified centers were contacted and invited to participate. Where legally necessary, enrolled patients or next of kin provided written informed consent, and the protocol was approved by relevant local ethics committees.

Academic investigators undertook the design, data collection, and analysis. The site investigators gathered the data, and monitoring and central pooling were performed by the central sites. The lead authors (T.R.M., D.W., T.Y.W., J.C.P., and D.J.S.) had unrestricted data access, and the analysis was performed by 2 independent clinician scientists (T.R.M. and D.W.). Background and details of the study design have been deposited previously (CRD42021277825), and the study is now closed to participation.

### Patients and Participating Centers

The study included patients from 64 centers worldwide recruited between 2008 and 2021. Most were tertiary care centers that offer IVT and mechanical thrombectomy 24 hours a day. Adult patients 18 years and older were eligible if they had an acute ischemic stroke, had confirmed ingestion of a DOAC within the last 48 hours, and underwent IVT (alteplase, dosage according to local guidelines, ie, 0.6 mg/kg bodyweight in Japan and 0.9 mg/kg bodyweight for all other centers; tenecteplase, 0.25 mg/kg dose only^27^). There was no limit with regard to time window for IVT. We excluded patients whose last known DOAC ingestion was more than 48 hours before stroke onset. No other exclusion criteria were applied, and centers were encouraged to provide records of consecutive patients over the study time frame. For the comparison group, we included patients who had ischemic stroke treated with IVT but received no prior anticoagulation therapy (ie, no VKA with INR greater than 1.7 or DOAC) from the prospective Thrombolysis in Ischemic Stroke Patients (TRISP) collaboration data set as well as participating centers.^28^ The control population was hence generated partially in a different time frame and contains data from few additional centers.

We divided patients into 2 mutually exclusive groups: (1) patients with confirmed last ingestion within the last 48 hours of any DOAC at a dose recommended for prevention of embolism in AF or treatment of pulmonary embolism or other indications (DOAC group) and (2) patients who used no anticoagulants (control group). Local investigators of the participating sites extracted from the local electronic health record information on the DOAC agent used before the event as well as its dosage, whether the time of last ingestion was documented, the time of last ingestion (categorized into within 12 hours, 12 to 24 hours, and more than 24 hours) as well as the exact time from last ingestion to admission in hours (if documented in sufficient detail). In some patients, the exact timing of DOAC therapy was unobtainable, but it was possible to assess the category (eg, a patient with aphasia presenting at noon unable to report the exact last ingestion time, but his wife verified that the blister of this morning was empty).

If the data were not already available from previously published data, local investigators at the participating centers conducted retrospective medical record reviews and local databank queries to collect standardized and prespecified variables using electronic case report forms. According to the availability at the specific site, this was done for both groups or for patients in the DOAC group only if there was no possibility to reliably identify controls. Data included patient demographic characteristics, medical history, antiplatelet treatment, clinical information, laboratory values, and information on recanalization treatments, including the use of IVT and mechanical thrombectomy as well as details on dose and workflow metrics. For patients with recent ingestion of DOACs treated with IVT, site investigators collected information on selection strategies, including DOAC-level measurements, specific DOAC reversal (idarucizumab or andexanet alfa) before IVT, receiving IVT but no measurement of DOAC plasma levels available or reversal, and receiving IVT inadvertently as the center did not know at the time that the patient was taking a DOAC. We collected information on availability of institutional guidelines or standard operating procedures (eTable 2 in Supplement 1) for IVT in patients with recent ingestion of DOACs and classified centers accordingly. In patients who received DOAC reversal, we assumed that this was the selection strategy, even if measurement of DOAC plasma level was available. Otherwise, if measurement of the DOAC plasma level was available, it was assumed that the decision for IVT included consideration of this information.

### Outcome Measures

The primary binary outcome was sICH defined as any intracranial hemorrhage as reported by the site investigators until 36 hours after IVT with associated neurological worsening of at least 4 points on the National Institutes of Health Stroke Scale (NIHSS) score compared with immediately before deterioration, attributed to the radiologically evident intracranial hemorrhage.^29^ Secondary outcomes included any radiological ICH as reported by the site investigators until 36 hours after IVT (whether accompanied by neurological worsening or not) and functional independence, defined as a modified Rankin Scale (mRS) score of 2 or less at 90 days and an ordinal regression analysis of the mRS. The mRS is a 7-point scale of global disability ranging from 0 (no symptoms) to 6 (death).^30^ It was assessed by site investigators during a clinical visit or a structured telephone interview.

### Statistical Analysis

The analysis followed a predefined analysis plan. We used medians with IQRs and means with SDs together with percentages to present the distribution of ordinal, continuous, and categorical variables. We compared baseline characteristics across groups using the Pearson χ^2^ test for categorical variables and *t* test or the Kruskal-Wallis test, as appropriate, for continuous and ordinal variables.

For the primary analysis, we used multilevel mixed-effects logistic regression analysis to compare odds of sICH between patients with recent ingestion of DOACs and controls with the geographic region included as a random effect. The rationale of the prespecified model was to adjust for known predictors of sICH.^31^ Those included age (continuous), arterial hypertension (yes or no), baseline NIHSS score (continuous), premorbid functional independence defined as a mRS score of 0 to 2 (yes or no), admission blood pressure (continuous), and admission glucose plasma level (continuous). To account for differences in local practices, we prespecified individual centers as a random effect in our models. However, because many centers had no sICH cases (which would exclude them from the model), we adjusted for geographic location as a random effect. We included recent DOAC ingestion as the independent variable, with controls as the reference group. We calculated adjusted odds ratios (ORs) and their corresponding 95% CIs. Because of missing data on this topic, no formal sample size calculation was possible; hence, a maximum of centers and patients was aimed for to ensure maximal precision.

To compare the different selection strategies (DOAC-level measurements, DOAC reversal before IVT, patients knowingly thrombolyzed but without measurement of DOAC plasma levels or reversal as well as inadvertent IVT with DOAC use subsequently discovered), the selection strategy was added to the model as a factor variable. We performed sensitivity analyses according to (1) geographic location (Asia, Europe, Australia, and New Zealand), (2) availability of institutional standard operating procedures, (3) patients with DOAC plasma levels greater than 100 ng/mL^24,32,33^ or proven ingestion within 12 hours before IVT, and (4) patients whose exact time of last DOAC ingestion was documented.

For the secondary outcomes, multilevel mixed-effects logistic and ordinal regression analysis were used to assess the association of recent DOAC ingestion with any ICH and functional outcome with the adjustments specified above. All analyses were performed by 2 clinician scientists (T.R.M. and D.W.) using Stata version 16 (StataCorp). All *P* values are 2-sided, with *P* < .05 considered statistically significant without adjustments for multiple testing. Complete case analysis was performed without imputations. Exploratory post hoc analyses are described as such.

## Results

### Study Enrollment and Characteristics of the Patients

We included 33 207 patients with acute ischemic stroke receiving IVT (832 who had taken a DOAC and 32 375 controls who did not take DOAC). Demographic and clinical characteristics of the patients at baseline are shown in Table 1. Of 33 207 included patients, 14 458 (43.5%) were female, and the median (IQR) age was 73 (62-80) years. The median (IQR) National Institutes of Health Stroke Scale score was 9 (5-16).

**Table 1.** Patient Baseline Characteristics

Characteristic	Total, No.	No. (%)			*P* value
		All patients (N = 33 207)	Controls (n = 32 375)	Patients with recent ingestion of DOACs (n = 832)	
Age, median (IQR), y	33 198	73 (62-80)	72 (62-80)	79 (71-85)	<.001
Sex					
Female	33 199	14 458 (43.5)	14 103 (43.6)	355 (42.7)	.60
Male		18 741 (56.5)	18 264 (56.4)	477 (57.3)	
Geographical region					
Europe	33 207	21 266 (64.0)	20 792 (64.2)	474 (57.0)	<.001
Asia		6698 (20.2)	6489 (20.0)	209 (25.1)	
Australia and New Zealand		5243 (15.8)	5094 (15.7)	149 (17.9)	
NIHSS score, median (IQR)^a^	32 928	9 (5-16)	9 (5-16)	11 (6-17)	<.001
Prestroke modified Rankin Scale score, median (IQR)^b^	29 059	0 (0-0)	0 (0-0)	0 (0-1)	<.001
Systolic blood pressure, mean (SD), mm Hg	29 178	154 (28)	154 (28)	154 (27)	.67
Time from symptom onset to hospital admission, median (SD), h	12 179	1.3 (0.8-2.3)	1.3 (0.8-2.3)	1.7 (1.0-2.6)	<.001
Blood glucose level, mean (SD), mg/dL	28 711	133 (48)	133 (48)	133 (45)	.77
International normalized ratio, median (IQR)	17 471	1 (1-1.1)	1 (1-1.1)	1.1 (1.02-1.2)	<.001
Risk factors and medication^c^					
Arterial hypertension	33 000	20 637 (62.5)	20 072 (62.2)	565 (75.1)	<.001
Current smoking	30 337	5891 (19.4)	5796 (19.6)	95 (12.8)	<.001
History of hypercholesterolemia	32 941	12 413 (37.7)	12 091 (37.6)	322 (43.2)	.002
Diabetes	32 978	6484 (19.7)	6311 (19.6)	173 (23.2)	.01
Antiplatelet therapy	29 884	10 471 (35.0)	10 383 (35.7)	88 (11.2)	<.001
AF	16 629	4616 (27.8)	4008 (25.1)	608 (90.1)	<.001
If taking anticoagulation, AF as reason for anticoagulation	1248	1171 (93.8)	609 (90.2)	562 (98.1)	<.001
Type of anticoagulation used					
Dabigatran	33 207	0	0	342 (41)	
Rivaroxaban		0	0	258 (31)	
Apixaban		0	0	163 (20)	
Edoxaban		0	0	68 (8)	
DOAC, not specified which agent		0	0	1 (<1)	
Vitamin K antagonists		0	689 (2.1)	0	
Presence of large vessel occlusion	33 069	10 970 (33.2)	10 516 (32.6)	454 (59.0)	<.001
Mechanical thrombectomy	32 833	6391 (19.5)	6106 (19.1)	285 (34.3)	<.001
Tenecteplase used (instead of alteplase)	33 199	1276 (3.8)	1225 (3.8)	51 (6.1)	<.001
Time from symptom onset to intravenous thrombolysis, median (IQR), min	29 717	138 (98-190)	138 (98-190)	153 (110-210)	<.001

Abbreviations: AF, atrial fibrillation; DOAC, direct oral anticoagulant; NIHSS, National Institutes of Health Stroke Scale.

SI conversion factor: To convert glucose to mmol/L, multiply by 0.0555.

^a^ Scores on the NIHSS range from 0 to 42, with 0 indicating no deficits and a higher score indicating more severe neurological deficits.

^b^ Score on the modified Rankin Scale range from 0 (no symptoms) to 6 (death). Prestroke disability was assessed by site investigators using information provided by the patient, health care records, and/or family members.

^c^ Risk factors denote known risk factors according to the medical history of the patient.

Compared with controls, patients with recent ingestion of DOACs were older, had a higher prevalence of hypertension, had a higher degree of prestroke disability, were less likely to be smokers, had a longer time from symptom onset to treatment, experienced more severe stroke, and were more likely to have a large vessel occlusion. Antiplatelet therapy was more frequent in controls. In patients receiving anticoagulation, AF was the most common indication. In selected centers that provided data on patients taking DOAC not receiving IVT (eTable 2 in Supplement 1), 19% (95% CI, 17-20) of IVT-eligible patients taking DOACs received IVT. The baseline comparison showed that those not receiving IVT were older, had a higher prevalence of vascular risk factors except smoking, had a higher degree of prestroke disability, had a longer time from symptom onset to admission, experienced less severe stroke, and were less likely to be treated with mechanical thrombectomy (eTable 3 in Supplement 1).

Of the patients taking DOACs, 355 (42.7%) were treated with IVT without measurement of DOAC plasma levels or administration of DOAC reversal, 252 (30.3%) received DOAC reversal prior to IVT (all idarucizumab among patients taking dabigatran), and for 225 (27.0%) of those selected, measurement of DOAC plasma levels was available. Information on latest ingestion, laboratory values, and details of acute recanalization treatment according to the selection strategies are presented in Table 2. Patients who had sICH differed significantly from those who did not (eTables 4 and 5 in Supplement 1).

**Table 2.** Details on Medication, Laboratory Workup, and Acute Recanalization Therapy According to the Selection Strategy Used

Measure	Total, No.	No. (%)			*P* value
		DOAC plasma levels measured (n = 225)	Neither known levels nor idarucizumab (n = 355)	Idarucizumab (n = 252)	
Age, median (IQR), y	832	80 (73-87)	79 (72-84)	77 (71-83)	.005
Sex					
Female	832	111 (49.3)	160 (45.1)	84 (33.3)	<.001
Male		114 (50.7)	195 (54.9)	168 (66.7)	
NIHSS score, median (IQR)	828	10 (6-16)	13 (7-18)	10 (6-16)	.006
Type of anticoagulation used					
Dabigatran	832	15 (6.7)	75 (21.1)	252 (100)	<.001
Rivaroxaban		119 (52.9)	139 (39.2)	0	
Apixaban		73 (32.4)	90 (25.4)	0	
Edoxaban		18 (8.0)	50 (14.1)	0	
DOAC agent not specified		0	1 (0.3)	0	
Time from last ingestion to admission					
<12 h	832	39 (17.3)	73 (20.6)	130 (51.6)	<.001
12-24 h		48 (21.3)	78 (22.0)	32 (12.7)	
24-48 h		43 (19.1)	59 (16.6)	1 (0.4)	
Exact time point unknown but <48 h		95 (42.2)	145 (40.8)	89 (35.3)	
Time from last ingestion to admission, median (IQR), h	503	14.4 (9-24)	14 (9.51667-25)	7 (4.75-11)	<.001
International normalized ratio, median (IQR)	674	1.1 (1-1.2)	1.1 (1.02-1.2)	1.13 (1.1-1.2)	.001
Activated partial thrombin time, median (IQR), s	664	29 (26-33)	30 (27-34)	37 (29-46)	<.001
Thrombin time, median (IQR), s	260	16.6 (15.2-18.3)	14.6 (11.4-17.4)	81.4 (43.9-120.0)	<.001
DOAC plasma level, median (IQR), ng/mL	244	21 (4.6-46)	NA	83 (27-134)	NA
Type of intravenous thrombolysis used					
Alteplase	831	223 (99.1)	351 (99.2)	206 (81.7)	<.001
Tenecteplase		2 (0.9)	3 (0.8)	46 (18.3)	
Time from symptom onset to intravenous thrombolysis, median (IQR), h	632	155 (105-230)	145 (97-190)	159 (120-202)	.03
Mechanical thrombectomy	832	79 (35.1)	139 (39.2)	67 (26.6)	.005
Time from symptom onset to groin puncture, median (IQR), h	199	188 (100-274)	182 (148-225)	296 (205-367)	<.001
Symptomatic intracranial hemorrhage within 36 h	832	7 (3.1)	11 (3.1)	3 (1.2)	.27
Any hemorrhagic transformation within 36 h	784	46 (20.5)	79 (22.3)	16 (7.8)	<.001

Abbreviations: DOAC, direct oral anticoagulant; NA, not applicable; NIHSS, National Institutes of Health Stroke Scale.

### Primary Outcome

Information on sICH was available for all 832 patients with recent ingestion of DOACs (100%) and 32 035 of 32 375 controls (98.9%). Overall, 1345 patients (4.1%; 95% CI, 3.9-4.3) developed sICH within 36 hours after IVT administration. The unadjusted rate of sICH was 2.5% (95% CI, 1.6-3.8) in patients taking DOACs compared with 4.1% (95% CI, 3.9-4.4) in control patients using no anticoagulants. After adjustment for stroke severity and other baseline sICH predictors, patients with recent ingestion of DOACs who received IVT had lower odds of developing sICH (adjusted OR, 0.57; 95% CI, 0.36-0.92; *P* = .02). There was no difference between the differing selection strategies (Table 3), and results were consistent in different sensitivity analyses (Table 4; eTable 6 in Supplement 1).

**Table 3.** Outcomes of Patients With Acute Ischemic Stroke Treated With Intravenous Thrombolysis by Selection Strategy

Outcome	Controls (n = 32 035)	All patients with recent ingestion of DOACs (n = 832)	DOAC plasma levels measured (n = 225)	Idarucizumab (n = 252)	Neither known levels nor idarucizumab (n = 355)
**Primary outcome**					
Symptomatic intracranial hemorrhage within 36 h, % (95% CI)	4.1 (3.9-4.4)	2.5 (1.6-3.8)	3.1 (1.3-6.3)	1.2 (0.2-3.4)	3.1 (1.6-5.5)
Unadjusted OR (95% CI)	NA	0.62 (0.40-0.96)	0.66 (0.31-1.40)	0.30 (0.09-0.92)	0.84 (0.46-1.53)
*P* value	NA	.03	.28	.04	.56
Adjusted OR (95% CI)	NA	0.57 (0.36-0.92)	0.56 (0.26-1.21)	0.36 (0.09-1.48)	0.66 (0.35-1.25)
*P* value	NA	.02	.14	.16	.20
**Secondary outcomes**					
Any hemorrhagic transformation on follow-up imaging within 36 h, % (95% CI)	17.4 (16.9-18.0)	18.0 (15.4-20.9)	20.5 (15.4-26.4)	7.8 (4.5-12.4)	22.2 (18.0-26.9)
Unadjusted OR (95% CI)	NA	1.03 (0.85-1.24)	1.23 (0.89-1.71)	0.38 (0.23-0.63)	1.40 (1.07-1.83)
*P* value	NA	.78	.21	<.001	.02
Adjusted OR (95% CI)	NA	1.18 (0.95-1.45)	1.13 (0.80-1.59)	0.57 (0.32-1.01)	1.58 (1.16-2.14)
*P* value	NA	.14	.49	.06	.003
Functional independence at 90 d, % (95% CI)	57 (56-57)	45 (41-49)	40 (33-47)	54 (46-62)	44 (38-50)
Unadjusted OR (95% CI)	NA	0.62 (0.53-0.73)	0.50 (0.37-0.67)	0.91 (0.66-1.25)	0.60 (0.48-0.74)
*P* value	NA	<.001	<.001	.55	<.001
Adjusted OR (95% CI)	NA	1.13 (0.94-1.36)	0.85 (0.61-1.19)	1.27 (0.84-1.91)	1.29 (0.99-1.68)
*P* value	NA	.20	.34	.26	.06

Abbreviations: DOAC, direct oral anticoagulant; NA, not applicable; OR, odds ratio.

**Table 4.** Prespecified Sensitivity Analyses

Subgroup	Total, No.	Association of DOAC intake with sICH <36 h, adjusted OR (95% CI)	*P* value
Geographical region			
Europe	474	0.60 (0.35-1.03)	.07
Asia	209	0.63 (0.23-1.74)	.37
Australia and New Zealand	149	0.34 (0.04-2.67)	.31
Standard Operating Procedure available at the study center			
Yes	729	0.54 (0.32-0.93)	.03
No	103	1.19 (0.37-3.83)	.77
Documented DOAC plasma levels >100 ng/mL or proven ingestion <12 h before IVT			
Yes	252	0.57 (0.23-1.42)	.23
No	580	0.57 (0.33-0.98)	.04

Abbreviations: DOAC, direct oral anticoagulant; IVT, intravenous thrombolysis; OR, odds ratio; sICH, symptomatic intracranial hemorrhage.

### Secondary Outcomes

Overall, 3724 patients (17.5%; 95% CI, 17.0-18.0) developed any ICH within 36 hours after IVT administration. The unadjusted rate of any ICH was 18.0% (95% CI, 15.4-20.9) in patients taking DOACs compared with 17.4% (95% CI, 16.9-18.0) in control patients who used no anticoagulants. After adjustment, there was no difference in the odds for any ICH between the groups (adjusted OR, 1.18; 95% CI, 0.95-1.45; *P* = .14).

Functional outcome at 90 days was known for 664 patients with recent ingestion of DOACs (79.8%) and 29 026 controls (87.4%) (eTables 7 and 8 in Supplement 1). Patients with missing outcome data had a similar prognostic profile. Overall, 16 765 patients (56.5%; 95% CI, 55.9-57.0) were functionally independent at 90 days. The unadjusted rate of functional independence was 45% (95% CI, 41-49) in patients taking DOACs compared with 57% (95% CI, 56-57) in control patients who used no anticoagulants. The unadjusted rate of death was 17.9% (95% CI, 15.1-21.1) in patients taking DOACs compared with 13.2% (95% CI, 12.8-13.6) in control patients who used no anticoagulants. After adjustment, patients with recent ingestion of DOACs who underwent IVT had numerically higher odds of being functionally independent than controls, although this difference did not reach statistical significance (adjusted OR, 1.13; 95% CI, 0.94-1.36; *P* = .20). There was no significant association of DOAC therapy with disability categories in the ordinal regression shift analysis (adjusted OR, 0.96; 95% CI, 0.84-1.11; *P* = .62), although the point estimate favored patients with recent ingestion of DOACs.

### Exploratory Post Hoc Analyses

The association of DOAC therapy with lower odds of sICH remained consistent when mechanical thrombectomy, large vessel occlusion, or concomitant antiplatelet therapy was added to the model (eTable 6 in Supplement 1). There was no clear difference in the primary outcome according to time from last DOAC ingestion or time window of IVT (eTables 9 and 10 in Supplement 1). There were no differences regarding the association of Xa inhibitors (adjusted OR, 0.59; 95% CI, 0.34-1.01; *P* = .06) and IIa inhibitors (adjusted OR, 0.54; 95% CI, 0.21-1.32; *P* = .18) with sICH. The rate of sICH among patients taking a VKA with an INR of 1.7 or less was 5.0% (95% CI, 3.5-6.9).

Regarding functional outcome, there was no difference between groups when mechanical thrombectomy was added to the models for the dichotomized functional independence (adjusted OR, 1.09; 95% CI, 0.90-1.32; *P* = .37) and the ordinal shift analysis (adjusted OR, 1.00; 95% CI, 0.86-1.15; *P* = .96). The odds of returning to the baseline mRS were similar between the 2 groups (OR, 1.02; 95% CI, 0.84-1.24; *P* = .84). There was no association of DOAC therapy and death at 3 months (adjusted OR, 0.97; 95% CI, 0.77-1.23; *P* = .82).

## Discussion

In this international, multicenter, retrospective cohort study of patients with acute ischemic stroke treated with IVT, the unadjusted rate of sICH was 2.5% (95% CI, 1.6-3.8) in patients taking DOACs compared with 4.1% (95% CI, 3.9-4.4) in control patients using no anticoagulants. After adjustment, recent use of DOACs (within 48 hours) was not associated with increased risk of sICH, regardless of whether patients were selected based on DOAC plasma levels, reversal with idarucizumab, or neither of those selection strategies.

A 2020 meta-analysis^34^ found that prior ingestion of DOACs appeared not to increase the risk of sICH in selected patients with ischemic stroke treated with IVT. Consistent with this observation, a 2022 analysis of the US-based Get With the Guidelines–Stroke Registry^2^ found no association of DOAC ingestion within the last 7 days before stroke onset and sICH. However, the major weakness in these studies was inclusion of only a small proportion of patients with verified ingestion within the past 48 hours, the time frame within which international guidelines recommend against IVT.^5,35^ The Get With the Guidelines report found that only 2 of 25 patients (8%) whose last DOAC ingestion was within 48 hours and none of 8 patients with last DOAC ingestion within 24 hours prior to hospital admission developed sICH.^2^ Additionally, details on selection strategies, including plasma level measurements, for these patients were not provided.^36^

Therefore, our study specifically addressed the patient population with documented last DOAC ingestion within the last 48 hours. IVT remains the cornerstone of recanalization therapies in patients with ischemic stroke, accompanied by mechanical thrombectomy, if indicated.^35,37,38^ Based on large retrospective observational studies like the current one, several of the former contraindications, including dual antiplatelet therapy,^39^ have been eliminated or have become less stringent (eg, IVT is now not a contraindication in patients who have recently had VKA treatment if their INR is less than 1.7^4,40^). Given the reduced risk of sICH in patients taking DOACs compared with VKA in the primary and secondary prophylaxis setting^1^ and supporting experimental data,^15,16,17,18^ it is therefore plausible that DOACs may not truly represent a contraindication to IVT after all. Given the established benefits of IVT regarding functional outcome, this analysis focused on safety, namely, the risk of sICH, a disabling and feared complication of IVT. However, this implies the assumption of a similar magnitude of benefit of IVT in patients taking DOACs.

We found a reduced risk of sICH in selected patients with recent ingestion of DOACs treated with IVT compared with controls, which is perhaps contrary to the perceived risk in this situation. In fact, the point estimates for all 3 selection strategies indicated lower odds of sICH compared with controls. One potential explanation for the low incidence of sICH in patients with recent ingestion of DOACs might be selection bias. This means that clinicians might only have offered IVT to patients with recent ingestion of DOACs who had a low probability of sICH, ie, earlier treatment window or no severe white matter hyperintensities. However, the time from symptom onset to treatment and patient characteristics (eg, older age) do not support this assumption. Second, residual and unmeasured confounding might explain the results observed. Although the lower risk of sICH seen in patients with recent ingestion of DOACs seems counterintuitive at first glance, there could be a pathophysiological explanation for this finding. In preclinical experimental stroke, no increased or even a purportedly lower risk of hemorrhage was found in animal models.^15,16,41^ Thrombin inhibition, either directly or via the coagulation cascade, might be protective against occurrence of sICH. This hypothesis is supported by a study that found no increased risk of sICH when IVT was combined with argatroban^42^ and is currently being tested in a clinical trial.^43^ The pathophysiological explanation for this might be that (low) DOAC concentrations could potentiate recanalization of the vessel occlusion and that thrombin inhibition could minimize disruption of the blood-brain barrier.^44^ Another explanation is that DOAC pretreatment might lead to smaller infarcts with lower risk of hemorrhagic transformation.^45^

This raises the question of how to proceed in the face of this dilemma in which a contraindication has been based on the eligibility criteria of the IVT trials but without sound preclinical or clinical evidence to support it. One possibility would be to perform a prospective randomized clinical trial to definitively assess the safety of IVT in patients with recent ingestion of DOACs, randomizing patients to receive IVT vs no IVT. In such a trial with a primary end point of sICH, determining a predefined upper limit of safety that is accepted by a large proportion of physicians, patients, and ethics committees would be challenging since such a trial in patients with recent ingestion of DOACs would have to enroll several thousand participants to ensure such precision. It is unlikely that such a trial will take place or be financed, and even if completed, it would not provide data for several years. We would also expect a strong inclusion bias, as patients qualifying for direct endovascular therapy would probably not be included.

With regard to systemic thrombolysis for other indications, physicians use fibrinolysis for acute myocardial infarction and pulmonary embolism, even using high doses in combination with intravenous heparin and list oral anticoagulation, including DOAC therapy, only as a relative contraindication.^46,47^ Given the established benefits of IVT and the absence of any signal for harm in our study or in other clinical studies or preclinical investigations, future guideline updates need to reconsider recent DOAC ingestion as a contraindication to IVT for acute ischemic stroke.

### Strengths and Limitations

The main strength of this study is that it specifically addresses patients taking DOACs with verified compliance and ingestion within 48 hours prior to hospital admission. On the other hand, the exact time point of ingestion was not known for a substantial portion of patients, and there were few patients with very recent ingestion. Further strengths are the perfect data completeness for the primary outcome in the DOAC group as well as almost perfect data quality for the control group.

Nevertheless, there are inherent limitations of the observational cohort design, including the risk of selection bias. Because of the nonrandomized setting, there were important baseline differences between patients with recent ingestion of DOACs and controls and potentially unmeasured confounding. The multilevel mixed-effect logistic regression might hence not have fully eliminated residual bias. Furthermore, the likely selection bias toward patients with a low probability for sICH seems especially relevant in the subgroup of patients who underwent IVT without measurement of DOAC plasma levels being known nor idarucizumab used prior to IVT. The control population was generated partially in a different time frame and with some additional centers compared with the DOAC group, and the data quality for patients taking DOACs not receiving IVT was low. The IVT dose given was somewhat heterogeneous since both Asian (reduced dose) and other centers worldwide contributed data. There were only 51 patients taking DOACs receiving tenecteplase (all 51 from New Zealand; 46 received idarucizumab); hence, more data on IVT in patients taking tenecteplase are needed. However, there was no signal of heterogeneity present according to the geographic region. Although no formal power calculation was possible, the study was probably underpowered to analyze multiple subgroups, including selection strategies, and the confidence intervals should hence be interpreted with caution. In the data set, information on systemic bleedings was not available, but the functional outcome data does not show evidence for harm overall. The baseline and follow-up imaging modality to assess hemorrhagic events was unavailable.

## Conclusions

In this international cohort study, we found insufficient evidence of excess harm associated with IVT in selected patients with ischemic stroke with recent DOAC ingestion. This was true regardless of the selection strategy and is likely consistent with net benefit of IVT in those patients.

## Notes

Supplement 1. **eTable 1.** Search Strategy PubMed/EMBASE **eTable 2.** Availability and Details of Standard Operating Procedures Regarding Intravenous Thrombolysis in Patients With Recent Ingestion of Direct Oral Anticoagulants **eTable 3.** Baseline Characteristics Comparing Patients With Preceding DOAC Prescription According to IVT Status in Selected Centers **eTable 4.** Patient Baseline Characteristics According to Occurrence of Symptomatic Intracranial Hemorrhage **eTable 5.** Patient Baseline Characteristics According to Occurrence of Symptomatic Intracranial Hemorrhage Limited to DOAC Patients **eTable 6.** Post Hoc Sensitivity Analyses for Outcomes of Patients With Acute Ischemic Stroke Treated With Intravenous Thrombolysis **eTable 7.** Rates of Outcome Categories in the Modified Rankin Scale at 90 Days According to Group **eTable 8.** Patient Baseline Characteristics According to Occurrence of Functional Independence (mRS Score of 0 to 2 at 90 Days) **eTable 9.** Rates of Symptomatic Intracranial Hemorrhage According to Time From Last Ingestion of a Direct Oral Anticoagulant **eTable 10.** Rates of Symptomatic Intracranial Hemorrhage According to Time From Symptom Onset to Needle According to DOAC Group

Supplement 2. Group Information. International DOAC-IVT, TRISP, and CRCS-K-NIH Collaboration

Supplement 3. Data Sharing Statement
