# Supplementary material for: Intravenous Thrombolysis in Patients With Ischemic Stroke and Recent Ingestion of Direct Oral Anticoagulants
Source: JAMA Neurol. 2023 Jan 3;80(3):233–43. doi: 10.1001/jamaneurol.2022.4782 (PMC9857462; doi:10.1001/jamaneurol.2022.4782)
Supplement: Supplement 1. — eTable 1. Search Strategy PubMed/EMBASE eTable 2. Availability and Details of Standard Operating Procedures Regarding Intravenous Thrombolysis in Patients With Recent Ingestion of Direct Oral Anticoagulants eTable 3. Baseline Characteristics Comparing Patients With Preceding DOAC Prescription According to IVT Status in Selected Centers eTable 4. Patient Baseline Characteristics According to Occurrence of Symptomatic Intracranial Hemorrhage eTable 5. Patient Baseline Characteristics According to Occurrence of Symptomatic Intracranial Hemorrhage Limited to DOAC Patients eTable 6. Post Hoc Sensitivity Analyses for Outcomes of Patients With Acute Ischemic Stroke Treated With Intravenous Thrombolysis eTable 7. Rates of Outcome Categories in the Modified Rankin Scale at 90 Days According to Group eTable 8. Patient Baseline Characteristics According to Occurrence of Functional Independence (mRS Score of 0 to 2 at 90 Days) eTable 9. Rates of Symptomatic Intracranial Hemorrhage According to Time From Last Ingestion of a Direct Oral Anticoagulant eTable 10. Rates of Symptomatic Intracranial Hemorrhage According to Time From Symptom Onset to Needle According to DOAC Group [file jamaneurol-e224782-s001.pdf]

Supplemental Online Content

Meinel TR, Wilson D, Gensicke H, et al; DOAC-IVT Writing Group; International DOAC-IVT, TRISP, and CRCS-K-NIH Collaboration. Intravenous thrombolysis in patients with ischemic stroke and recent ingestion of direct oral anticoagulants. *JAMA Neurol*. Published online January 3, 2023. doi:10.1001/jamaneurol.2022.4782

eTable 1. Search Strategy PubMed/EMBASE

eTable 2. Availability and Details of Standard Operating Procedures Regarding Intravenous Thrombolysis in Patients With Recent Ingestion of Direct Oral Anticoagulants

eTable 3. Baseline Characteristics Comparing Patients With Preceding DOAC Prescription According to IVT Status in Selected Centers

eTable 4. Patient Baseline Characteristics According to Occurrence of Symptomatic Intracranial Hemorrhage

eTable 5. Patient Baseline Characteristics According to Occurrence of Symptomatic Intracranial Hemorrhage Limited to DOAC Patients

eTable 6. Post Hoc Sensitivity Analyses for Outcomes of Patients With Acute Ischemic Stroke Treated With Intravenous Thrombolysis

eTable 7. Rates of Outcome Categories in the Modified Rankin Scale at 90 Days According to Group

eTable 8. Patient Baseline Characteristics According to Occurrence of Functional Independence (mRS Score of 0 to 2 at 90 Days)

eTable 9. Rates of Symptomatic Intracranial Hemorrhage According to Time From Last Ingestion of a Direct Oral Anticoagulant

eTable 10. Rates of Symptomatic Intracranial Hemorrhage According to Time From Symptom Onset to Needle According to DOAC Group

This supplementary material has been provided by the authors to give readers additional information about their work.

© 2023 Meinel TR et al. *JAMA Neruology*.

eTable 1. Search Strategy PubMed/EMBASE

**Concept 1 (ischemic stroke)**	"Stroke"[Mesh] OR<br>"Cerebrovascular Disorders"[MeSh] OR "Intracranial Embolism and Thrombosis"[Mesh]<br><br>OR<br><br>"stroke"[title/abstract] OR<br>ischemic stroke[Title/Abstract] OR<br>brain infarction*[Title/Abstract]
**AND**	
**Concept 2 (DOAC therapy)**	"Anticoagulants" [Mesh]<br>OR<br><br>"DOAC" "[title/abstract] OR<br>"NOAC"[title/abstract] OR<br>"direct oral anticoag*"[title/abstract] OR<br>"novel oral anticoag*"[title/abstract] OR<br>"anticoagul*"[title/abstract] OR<br>"rivaroxaban"[title/abstract] OR<br>"dabigatran"[title/abstract] OR<br>"apixaban"[title/abstract] OR<br>"edoxaban"[title/abstract] OR<br>"idarucizumab"[title/abstract]
**AND**	
**Concept 3 (intravenous thrombolysis)**	"Thrombolytic Therapy" [Mesh]<br>OR<br>"thrombolysis" [Title/Abstract] OR<br>"thrombolytics" [Title/Abstract] OR<br>"tPA" Title/Abstract] OR<br>"tissue plasminogen activator" [Title/Abstract] OR<br>"thrombolytics" [Title/Abstract] OR<br>"rt-PA" [Title/Abstract] OR<br>"Alteplase" [Title/Abstract] OR<br>"Tenecteplase" [Title/Abstract]
**Filters**: (("2000/01/01"[PDAT] : "2021/31/07"[PDAT])<br>AND<br>"humans"[MeSH Terms] AND<br>(English[lang]))	

© 2023 Meinel TR et al. *JAMA Neruology*.

eTable 2. Availability and Details of Standard Operating Procedures Regarding Intravenous Thrombolysis in Patients With Recent Ingestion of Direct Oral Anticoagulants

Center	Number of DOAC-IVT cases	Number of IVT controls (without DOAC)	Number of DOAC cases not receiving IVT	Details on Standard Operating Procedure
Adelaide	29	850	175	thrombolyse without levels if >24 hours since ingestion, idarucizumab without waiting for levels if dabigatran ingestion known or suspected <24 hours and apixaban/rivaroxaban levels guiding thrombolysis if known or suspected ingestion <24 hours.
Akershus	20	988	49	Dabigatran < 12 hours since last intake and not possible to reverse. Other DOACs - <24 hours since last intake. Relative contraindications Dabigatran, IVT may be given if > 12 hours and normal APTT and INR IVT may be given if reversal with anti-dot is possible. Other DOACs - IVT may be given if 24-48 hours since last intake and normal kidney function. (Decision also supported by normal INR, Thrombocytes count, APTT)
Amsterdam	11	1,236		
Auckland	10	0		Idarucizumab + Rivaroxaban Levels
Basel	31	1,607	463	DOAC level based
Belgrad	0	501		Obtaining the data of DOAC intake for > 48 h or in case of < 48h - normal laboratory tests such as aPTT, INR, platelet count. The analysis of direct factor Xa activity has recently been introduced. The detection of DOAC plasma levels has not yet been introduced in our center's SOP.
Berlin	17	1,843	438	If certain intake of DOACs > 24 hours and CrCL>50ml: IVT possible (information about off-label IVT use). If anamnesis unclear or intake of DOACs (other than dabigatran) < 24 h: IVT if INR (< 1.4), TZ and PT (Quick) are normal. Before praxbind available: If on dabigatran, aPTT above normal range OR medication ingested within 24 hours prior to lab drawn: no IVT. After praxbind available: if certain intake of dabigatran <24h IVT possible after administration of Praxbind 5g (information about off-label IVT use).
Bern	30	1,466	456	DOAC level based
Bologna	24	316		DOAC level based
Box Hill	0	3		At Box Hill, we do acute DOAC levels, and use cut-off as suggested in the Australian Stroke Guideline: dabigatran < 40 ng/mL, apixaban < 10 ng/mL and rivaroxaban < 100 ng/mL (https://informme.org.au/Guidelines/Clinical-Guidelines-for-Stroke-Management).<br><br>Dabigatran --> praxbind and Xa threshold of <100ng/ml to give

© 2023 Meinel TR et al. *JAMA Neruology*.

Brescia	1	388		DOAC level and reversal
Christchurch	40	639		Only reversal with Idarucizumab possible
Dijon	5	408		DOAC plasma levels in first line using the cut-off of 50 ng/ml (up to 100 ng/mL in some patients), according to the 2016 French Guideline
Erlangen	43	0	334	DOAC level based and reversal with idarucizumab
Frankfurt Hoechst	0	2		For dabigatran > 30 ng/ml: idaruzicumab, thrombolysis, thrombectomy (if necessary and no CI); For FXa inhibitor > 30 ng/ml: no lysis, thrombectomy (if no contraindications)
Fuijan	1	364		Only if Idarucizumab possible, no DOAC levels
German Multicenter	80	0		only Dabigatran cases (after availaibility of Idarucizumab)
Gothenburg	0	86		
Heidelberg	41	2,849	339	Dabigatran: idarucizumab; all: DOAC level based
Helsinki	6	3,553		Dabigatran: Idarucizumab, other DOACs: measurement of the activity, lab results within 20 mins. no use of andexanet
Hong Kong	4	94	29	We exclude patient with last DOAC intake within 48 hours from IVT<br>For those with last intake within 48 hours or unknown onset time, we perform CTA and proceed to EVT for those with LVO<br>Hence included patients erroneous IVT.
Hospital Joan XXIII	0	2		DOAC reversal, no DOAC levels
Hospital Universitari Bellvitge (HUB)	0	1		
Japan Multicenter	35	70		
Jerusalem	7	341		We measure plasma DOAC level and XA or TT levels and we do have access to both andexanet and praxbind. We do not use reversal agents if TT and PTT are normal (for dabigatran) or if Xa is less thean 0.5 and low plasma levels for apixaban or rivaroxaban
John Hunter	4	144	107	
Kansai	9	139		
Korea multicenter	82	5,857	378	
LMU Munich	21	0		
Larissa	4	36		
Lausanne	30	1,075	293	DOAC level based
Lille	4	1,515		DOAC level based and reversal with idarucizumab
Lisbon	4	0		IVT contraindicated if anti-Xa taken <48h; If anti-IIa taken <48h-->first option direct MT; if not eligible for MT, IVT after reversal with idarucizumab. At the moment: if anti-Xa or IIa taken <48h--->first option direct MT; if not eligible

© 2023 Meinel TR et al. *JAMA Neruology*.

				for MT: IVT if anti-Xa< 50ng/ml or anti-IIa with TT<60s; if anti-II with TT>60s IVT after reversal with idarucizumab; IVT after reversal with andexanet alfa not considered.
Lugano	10	126	159	
Mainz	3	0	87	DOAC plasma levels + reversal
Modena	4	1,288		
München/TUM	6	0		Only if Idarucizumab possible
Osaka	41	0		Japanese guidelines
Paris	4	0		
Perugia	7	0		Advised performing plasma levels on all people with stroke taking DOAC with last intake >2 hours before, reversal advised in case of dabigatran. No threshold defined for thrombolysis with other DOACs. If DOAC taken <2h no test advised, no IVT as for recommendations.
Reggio Emilia	7	578		DOAC plasma levels
Royal Melbourne	18	866		
St. Gallen	28	201		Depends on anti-Xa activity (100ng/ml) or thrombin time <20sec in case of dabigatran treatment. No use of reversal agents before IVT so far.
Tübingen	8	188		DOAC level/POC INR
UCLH	2	0	34	
UKE Hamburg	10	0		Dabigatran with idarucizumab. DOAC level/activity measurements in our SOP for lysis not provided --> no lysis for Xa inhibitors <48h
Umbria	1	0		
Wellington	45	2,595		If Dabigatran within last 48 hours and/or abnormal TT give 5g Idarucizumab. Wait 5min treat. If Rivaroxaban/Apixaban within last 48 hrs don't thrombolyse. Warfarin needs an INR <1.7z
Zürich	2	203	296	If medication is taken certainly >4 hours before lab and normal kidney function, lysis is justifiable with benefit-risk assessment (off-label): a. Rivaroxaban: anti-Factor Xa activity (Rivaroxaban) <100 µg/L Can be determined in the emergency laboratory and without consulting of Haematology. Lysis acceptable with anti-Factor Xa activity (Rivaroxaban) <100 µg/L. b. Apixaban: anti-factor Xa activity (LMWH) normal (=0), with slightly increased values in consultation with hematology lysis decision based on this; also always anti-factor Xa activity (Apixaban) set (approx. 1.5h). Lysis acceptable with anti-factor Xa activity (apixaban) <40 µg/l. c. Dabigatran: thrombin time normal or moderate after individual benefit-risk assessment Thrombin time prolongation (<4x upper limit of normal). In consultation with hematology lysis decision based on this; also always allow anti-factor IIa activity

© 2023 Meinel TR et al. *JAMA Neruology*.

				dabigatran to develop (approx. 1.5 h). Lysis acceptable with anti-factor IIa activity (dabigatran) <50 ng/ml (<0.05 µg/ml). at Dabigatran levels >0.05 µg/ml (>50 ng/ml) or medication intake <4h can lead to lysis immediately preceding antagonism with idarucizumab (Praxbind® , standard dose 5 g IV) be considered. i.e. Edoxaban: anti-factor Xa activity (LMWH) normal (=0), with slightly increased values in RS with YES hematology lysis decision based on this; also always anti-factor Xa activity Allow edoxaban to set in (approx. 1.5 hours). A specific anti-Fxa test is now available for edoxaban Disposal; there are still no recommendations regarding a limit value for the lysis test. e. Unknown anticoagulant Quick/INR, thrombin time, anti-factor Xa activity (LMWH) normal → not relevant Plasma concentration of VKA, heparins, anti-FXa or thrombin antagonists f. VKA antagonists (Marcoumar, Sintrom, Warfarin): Lysis with rtPA acceptable from INR ≤1.7. Note: • Considerable delay in DOAC elimination possible in the case of renal insufficiency • If blood is taken very shortly after ingestion (<2-4 hours), the maximum Adjust plasma levels of a DOAC only after blood collection
			The difference between the number of study centers in the manuscripts and listed here is due to the inclusion of multicenter cohorts.	
	832	32,375		

© 2023 Meinel TR et al. *JAMA Neruology*.

eTable 3. Baseline Characteristics Comparing Patients With Preceding DOAC Prescription According to IVT Status in Selected Centers

	DOAC patients not receiving IVT (N= 3,603)	Patients with recent ingestion of DOACs receiving IVT (N=348)	*P*
Median age – yrs (SD)	81 (75-86)	78 (72-85)	<0.001
Female sex – yes (%)	1771 (49%)	164 (47%)	0.47
Median NIHSS score (IQR) [a]	7 (3-15)	10 (6-17)	<0.001
Median pre-stroke score on the modified Rankin Scale (IQR) [b]	1 (0-2)	1 (0-1)	<0.001
Mean systolic blood pressure – mmHg (SD)	154 (28)	156 (29)	0.33
Median time from symptom onset to hospital admission – h (IQR)	2.7 (1.3-7.1)	1.6 (1-2.9)	<0.001
Mean blood glucose level – mmol/L (SD)	7.8 (3.1)	7.4 (2.6)	0.045
Median international normalized ratio – (IQR)	1.3 (1.1-1.8)	1.1 (1.02-1.2)	<0.001
**Risk factors and medication**[c]			
Arterial hypertension – yes (%)	3111 (86%)	278 (80%)	<0.001
Current smoking – yes (%)	292 (8.5%)	41 (11.9%)	0.03
History of hypercholesterolemia – yes (%)	1991 (56%)	174 (50%)	0.046
Diabetes Mellitus – yes (%)	1050 (29%)	98 (28%)	0.75
Presence of large vessel occlusion – yes (%)	1617 (45%)	201 (60%)	<0.001
Mechanical thrombectomy – yes (%)	878 (27%)	135 (39%)	<0.001
Restricted to centers that provided both DOAC patients that underwent and did not undergo intravenous thrombolysis.			

© 2023 Meinel TR et al. *JAMA Neruology*.

eTable 4. Patient Baseline Characteristics According to Occurrence of Symptomatic Intracranial Hemorrhage

	N available	No symptomatic Intracranial Hemorrhage (N=31,522)	Symptomatic Intracranial Hemorrhage (N=1,345)	*P*
Median age – yrs (IQR)	32,859	72 (62-80)	75 (67.7-82)	<0.001
Female sex – yes (%)	32,860	13,710 (43.5%)	609 (45.3%)	0.20
Geographical region	32,867			<0.001
Europe – no. (%)		19,970 (63.4%)	992 (73.8%)	
Asia – no. (%)		6,500 (20.6%)	198 (14.7%)	
Australia/New Zealand – no. (%)		5,052 (16.0%)	155 (11.5%)	
Median NIHSS score (IQR) [a]	32,593	9 (5-15)	13 (8-18)	<0.001
Median pre-stroke score on the modified Rankin Scale (IQR) [b]	28,758	0 (0-0)	0 (0-0)	<0.001
Mean systolic blood pressure – mmHg (SD)	28,922	154 (28)	158 (27)	<0.001
Mean blood glucose level – mmol/L (SD)	28,459	7.38 (2.66)	8.06 (3.20)	<0.001
Median international normalized ratio – (IQR)	17,285	1 (1-1.1)	1 (1-1.1)	0.009
**Risk factors and medication**[c]				
Arterial hypertension – yes (%)	32,739	19,553 (62.3%)	929 (69.4%)	<0.001
Current smoking – yes (%)	30,219	5,687 (19.6%)	174 (15.3%)	<0.001
History of hypercholesterolemia – yes (%)	32,682	11,827 (37.7%)	509 (38.3%)	0.69
Diabetes Mellitus – yes (%)	32,717	6,109 (19.5%)	326 (24.3%)	<0.001
Antiplatelet therapy – yes/no. (%)	29,624	9,824 (34.6%)	554 (44.9%)	<0.001

© 2023 Meinel TR et al. *JAMA Neruology*.

Presence of large vessel occlusion – yes (%)	32,729	10,343 (33.0%)	561 (41.7%)	<0.001
Mechanical thrombectomy – yes (%)	32,495	6,032 (19.4%)	318 (23.7%)	<0.001
Vitamin K antagonists	32,867	647 (2.1%)	34 (2.5%)	0.23
Recent ingestion of direct oral anticoagulants	32,867	811 (2.6%)	21 (1.6%)	0.021
Direct oral anticoagulants, plasma level ng/ml	244	23 (7.5-48.98)	18 (4.59-55.5)	0.80
Time from symptom onset to needle, min	29,458	138 (98-190)	143 (104-194)	0.024

© 2023 Meinel TR et al. *JAMA Neruology*.

eTable 5. Patient Baseline Characteristics According to Occurrence of Symptomatic Intracranial Hemorrhage Limited to DOAC Patients

	N available	No symptomatic Intracranial Hemorrhage (n= N=811)	Symptomatic Intracranial Hemorrhage (N=21)	*P*
Mean age – yrs (SD)	832	78 (71-85)	80 (74-84)	0.39
Female sex – yes (%)	832	345 (42.5%)	10 (47.6%)	0.64
Geographical region	832			0.54
Europe – no. (%)		460 (56.7%)	14 (66.7%)	
Asia – no. (%)		204 (25.2%)	5 (23.8%)	
Australia/New Zealand – no. (%)		147 (18.1%)	2 (9.5%)	
Median NIHSS score (IQR) [a]	828	11 (6-17)	11 (9-15)	0.58
Median pre-stroke score on the modified Rankin Scale (IQR) [b]	749	0 (0-1)	1 (0-1.5)	0.49
Mean systolic blood pressure – mmHg (SD)	746	154.117 (26.6421)	149.333 (25.4093)	0.42
Mean blood glucose level – mmol/L (SD)	745	7.38562 (2.52458)	7.04648 (1.44313)	0.55
Median international normalized ratio – (IQR)	674	1.1 (1.02-1.2)	1.1 (1-1.2)	0.91
**Risk factors and medication**[c]				
Arterial hypertension – yes (%)	752	552 (75.5%)	13 (61.9%)	0.15
Current smoking – yes (%)	742	94 (13.0%)	1 (5.0%)	0.29
History of hypercholesterolemia – yes (%)	745	312 (43.1%)	10 (47.6%)	0.68
Diabetes Mellitus – yes (%)	746	168 (23.2%)	5 (23.8%)	0.95
Antiplatelet therapy – yes/no. (%)	787	84 (11.0%)	4 (19.0%)	0.25
Presence of large vessel occlusion – yes (%)	770	440 (58.7%)	14 (66.7%)	0.47
Mechanical thrombectomy – yes (%)	832	273 (33.7%)	12 (57.1%)	0.025

© 2023 Meinel TR et al. *JAMA Neruology*.

Direct oral anticoagulants, plasma level ng/ml	244	23 (7.5-48.98)	18 (4.59-55.5)	0.80
Low Dose alteplase	781	204 (26.8%)	5 (23.8%)	0.76
Time from symptom onset to needle, min	632	154.5 (111.5-210)	111.5 (74.5-215)	0.14

© 2023 Meinel TR et al. *JAMA Neruology*.

eTable 6. Post Hoc Sensitivity Analyses for Outcomes of Patients With Acute Ischemic Stroke Treated With Intravenous Thrombolysis

	Unadjusted rate DOAC group with confidence interval	Unadjusted rate control group with confidence interval	Adjusted Odds Ratio of association of recent DOAC ingestion with symptomatic intracranial hemorrhage
Excluding control cases from prior to the collection period of DOAC cases (before 2013)	2.5% (1.6–3.8), N=832	4.1% (3.8–4.3), N=29,732	0.57 (0.36-0.91) P=0.019
Excluding VKA patients (with an INR <1.7) from the control group	2.5% (1.6–3.8), N=832	4.1% (3.9–4.3), N= 31,354	0.57 (0.36-0.91) P=0.02
Adding mechanical thrombectomy to the model			0.54 (0.34-0.87) P=0.01
Adding large-vessel occlusion to the model			0.55 (0.35-0.88) P=0.01
Adding concomitant antiplatelet therapy to the model			0.65 (0.41-1.04) P=0.072
Adding atrial fibrillation to the model			0.46 (0.27-0.81) P=0.007
Restricted to patients with documented time from last ingestion to admission		Combined (n=503)<br><br><12h (n=242)<br><br>12-24h (n=158)<br><br>24-48h (n=103)	0.62 (0.35-1.1), P=0.11<br>0.59 (0.24-1.47), P=0.26<br>0.45 (0.14-1.41), P=0.17<br>0.93 (0.34-2.56), P=0.892
Documented DOAC plasma levels >100ng/ml or proven ingestion <12 hours before IVT and without Idarucizumab		Yes (n=118)<br><br>No (n=714)	0.69 (0.25-1.9), P=0.472<br>0.55 (0.33-0.93), P=0.025
Results show the rate (with 95% confidence interval) and the adjusted odds ratio in the fully adjusted model for the association of recent ingestion of a direct oral anticoagulant with the primary outcome (symptomatic intracranial hemorrhage)			

© 2023 Meinel TR et al. *JAMA Neruology*.

eTable 7. Rates of Outcome Categories in the Modified Rankin Scale at 90 Days According to Group

Modified Rankin Scale Category	Patients with recent ingestion of DOACs (n= 664)	Controls (n= 29,026)
0	89 (13.4%)	5,861 (20.2%)
1	106 (16.0%)	5,963 (20.5%)
2	104 (15.7%)	4,642 (16.0%)
3	110 (16.6%)	3,998 (13.8%)
4	82 (12.4%)	3,103 (10.7%)
5	54 (8.1%)	1,623 (5.6%)
6	119 (17.9%)	3,836 (13.2%)

© 2023 Meinel TR et al. *JAMA Neruology*.

eTable 8. Patient Baseline Characteristics According to Occurrence of Functional Independence (mRS Score of 0 to 2 at 90 Days)

	N available	Poor Outcome at 90 days mRS 3-6 (N=12,925)	Functional Independence at 90 days mRS 0-2 (N=16,765)	*P*
Median age – yrs (IQR)	29,687	77 (68-84)	69 (58-77)	<0.001
Female sex – yes (%)	29,685	6,457 (50.0%)	6,520 (38.9%)	<0.001
Geographical region	29,690			0.33
Europe – no. (%)		8,755 (67.7%)	11,277 (67.3%)	
Asia – no. (%)		2,603 (20.1%)	3,359 (20.0%)	
Australia/New Zealand – no. (%)		1,567 (12.1%)	2,129 (12.7%)	
Median NIHSS score (IQR) [a]	29,581	14 (8-19)	6 (4-11)	<0.001
Median pre-stroke score on the modified Rankin Scale (IQR) [b]	27,206	0 (0-1)	0 (0-0)	<0.001
Mean systolic blood pressure – mmHg (SD)	26,977	155 (30.9)	154 (25.6)	0.002
Mean blood glucose level – mmol/L (SD)	26,656	7.8 (2.9)	7.1 (2.4)	<0.001
Median international normalized ratio – (IQR)	16,659	1.02 (1-1.1)	1 (.99-1.1)	<0.001
**Risk factors and medication**[c]				
Arterial hypertension – yes (%)	29,627	9,045 (70.2%)	10,058 (60.1%)	<0.001
Current smoking – yes (%)	27,154	1,799 (15.4%)	3,640 (23.5%)	<0.001
History of hypercholesterolemia – yes (%)	29,572	4,809 (37.4%)	6,683 (40.0%)	<0.001
Diabetes Mellitus – yes (%)	29,605	3,154 (24.5%)	2,788 (16.7%)	<0.001
Antiplatelet therapy – yes/no. (%)	27,570	4,777 (39.6%)	5,072 (32.7%)	<0.001

© 2023 Meinel TR et al. *JAMA Neruology*.

Presence of large vessel occlusion – yes (%)	29, 566	5,368 (41.8%)	4,749 (28.4%)	<0. 00 1
Mechanical thrombectomy – yes (%)	29, 318	3,100 (24.2%)	2,761 (16.7%)	<0. 00 1
Time from symptom onset to needle, min	26, 746	140 (100-188)	135 (95-186)	<0. 00 1

© 2023 Meinel TR et al. *JAMA Neruology*.

eTable 9. Rates of Symptomatic Intracranial Hemorrhage According to Time From Last Ingestion of a Direct Oral Anticoagulant

**Time from last ingestion of DOAC**	**All patients**	**DOAC plasma levels measured**	**Neither known levels nor Idarucizumab**	**Idarucizumab**
<12 h	2.9%, 1.1-5.9%	5.1%, 0.6-17.3%	4.1%, 0.9-11.5%	1.5%, 0.02-5.4%
12–24 h	1.9%, 0.4-5.4%	2.1%, 0.01-11.1%	1.3%, 0.01-6.9%	3.1, 0.01-16.2%
24–48 h	3.9%, 1.1-9.6%	2.3%, 0.01-12.3%	5.1%, 1.1-14.1%	0%, 0-98%
Exact time point unknown, but certainly <48 h	2.1%, 0.9-4.3%	3.2%, 0.01-9.0%	1.3%, 0.8-6.9%	0%, 0-4.1%
	Symptomatic intracranial hemorrhage %, 95% CI			

© 2023 Meinel TR et al. *JAMA Neruology*.

eTable 10. Rates of Symptomatic Intracranial Hemorrhage According to Time From Symptom Onset to Needle According to DOAC Group

	**DOAC**	**Controls**
**0-3h**	sICH 2.8%, 1.5-4.9%, n=422	sICH 3.8%, 3.5-4.1%, N= 20,898
**3-4.5h**	sICH 0.6%, 0.0-3.6%, n=51	sICH 3.9%, 3.5-4.5%, N= 6,507
**extended window/wake-up**	sICH 3.1%, 1.3-6.0%, n=258	sICH 4.3%, 3.3-5.5%, N= 1,421

© 2023 Meinel TR et al. *JAMA Neruology*.
