# Supplementary material for: Intravenous Thrombolysis in Patients With Ischemic Stroke and Recent Ingestion of Direct Oral Anticoagulants
Source: JAMA Neurol. 2023 Jan 3;80(3):233–43. doi: 10.1001/jamaneurol.2022.4782 (PMC9857462; doi:10.1001/jamaneurol.2022.4782)
Supplement: Supplement 2. — Group Information. International DOAC-IVT, TRISP, and CRCS-K-NIH Collaboration [file jamaneurol-e224782-s002.pdf]

Supplemental Online Content: Nonauthor Collaborators
*First name, last name, and suffix (if applicable) are required and will appear in PubMed.

*Group Name(s): International DOAC-IVT, TRISP, and CRCS-K-NIH Collaboration							
***First Name and Middle Initial(s)**	***Last Name**	***Suffix (eg, Jr, III)**	Academic Degrees	Institution	Location (city, state/province, country)	Role or Contribution, eg, chair, principal investigator	Group (if more than 1 Group listed in the byline) and/or Subgroup (eg, Steering Committee)
Martina	Goeldlin		MD	Department of Neurology, Inselspital, Bern University Hospital, and University of Bern, Switzerland.			DOAC-IVT
Bernhard	Siepen		MD	Department of Neurology, Inselspital, Bern University Hospital, and University of Bern, Switzerland.			DOAC-IVT
Madlaine	Mueller		MD	Department of Neurology, Inselspital, Bern University Hospital, and University of Bern, Switzerland.			DOAC-IVT
Morin	Beyeler		MD	Department of Neurology, Inselspital, Bern University Hospital, and University of Bern, Switzerland.			DOAC-IVT
Adrian	Scutelnic		MD	Department of Neuroradiology, Inselspital, Bern University Hospital, and University of Bern, Switzerland.			DOAC-IVT
Simon	Jung		MD	Department of Neurology, Inselspital, Bern University Hospital, and University of Bern, Switzerland.			DOAC-IVT
Adnan	Mujanovic		MD	Department of Neuroradiology, Inselspital, Bern University Hospital, and University of Bern, Switzerland.			DOAC-IVT
Christoph	Kurmann		MD	Department of Neuroradiology, Inselspital, Bern University Hospital, and University of Bern, Switzerland.			DOAC-IVT

Supplemental Online Content: Nonauthor Collaborators
*First name, last name, and suffix (if applicable) are required and will appear in PubMed.

*First Name and Middle Initial(s)	*Last Name	*Suffix (eg, Jr, III)	Academic Degrees	Institution	Location (city, state/province, country)	Role or Contribution, eg, chair, principal investigator	Group (if more than 1 Group listed in the byline) and/or Subgroup (eg, Steering Committee)
Tomas	Dobrocky		MD	Department of Neuroradiology, Inselspital, Bern University Hospital, and University of Bern, Switzerland.			DOAC-IVT
Eike	Piechoviak		MD	Department of Neuroradiology, Inselspital, Bern University Hospital, and University of Bern, Switzerland.			DOAC-IVT
Roland	Wiest		MD	Department of Neuroradiology, Inselspital, Bern University Hospital, and University of Bern, Switzerland.			DOAC-IVT
Arsany	Hakim		MD	Department of Neuroradiology, Inselspital, Bern University Hospital, and University of Bern, Switzerland.			DOAC-IVT
Philipp	Bücke		MD	Department of Neurology, Inselspital, Bern University Hospital, and University of Bern, Switzerland.			DOAC-IVT
Zarzitzky	Jana		MD	Department of Neurology, Klinikum Frankfurt Höchst, Germany			TRISP
Lichti	Carmen		MD	Department of Neurology, Klinikum Frankfurt Höchst, Germany			TRISP
Dmytrow	Diana Isabella		MD	Clinical Study Coordination Office, Klinikum Frankfurt Höchst, Germany			TRISP
João Nuno	Ramos		MD	Deparment of Neuroradiology, Hospital de Egas Moniz, Centro Hospitalar Lisboa Ocidental, Lisbon Portugal			TRISP

Supplemental Online Content: Nonauthor Collaborators
*First name, last name, and suffix (if applicable) are required and will appear in PubMed.

*First Name and Middle Initial(s)	*Last Name	*Suffix (eg, Jr, III)	Academic Degrees	Institution	Location (city, state/province, country)	Role or Contribution, eg, chair, principal investigator	Group (if more than 1 Group listed in the byline) and/or Subgroup (eg, Steering Committee)
Davide	Strambo		MD	Department of Neurology, Service of Clinical Neurosciences, Lausanne University Hospital and University of Lausanne, Lausanne, Switzerland.			TRISP
Menuela	Fyilviv		MD	Department of Neurology, Service of Clinical Neurosciences, Lausanne University Hospital and University of Lausanne, Lausanne, Switzerland.			TRISP
Naaem	Simaan		MD	Department of Neurology, Hadassah-Hebrew University Medical Center			TRISP
Asaf	Honig		MD	Department of Neurology, Hadassah-Hebrew University Medical Center			TRISP
Andrei	Filioglo		MD	Department of Neurology, Hadassah-Hebrew University Medical Center			TRISP
Ilaria	Grisendi		MD	Neurology Unit, Stroke Unit, Azienda Unità Sanitaria Locale - IRCCS di Reggio Emilia, Italy			TRISP
Laura	Ferri		MD	Neurology Unit, Stroke Unit, Azienda Unità Sanitaria Locale - IRCCS di Reggio Emilia, Italy			TRISP
Maria Claudia	Trapasso		MD	Neurology Unit, Stroke Unit, Azienda Unità Sanitaria Locale - IRCCS di Reggio Emilia, Italy			TRISP
Matteo	Benini		MD	Neurology Unit, Stroke Unit, Azienda Unità Sanitaria Locale - IRCCS di Reggio Emilia, Italy			TRISP
Rosario	Pascarella		MD	Neuroradiology Unit, Azienda Unità Sanitaria Locale - IRCCS di Reggio Emilia, Italy			TRISP

*First name, last name, and suffix (if applicable) are required and will appear in PubMed.

***First Name and Middle Initial(s)**	***Last Name**	***Suffix (eg, Jr, III)**	Academic Degrees	Institution	Location (city, state/province, country)	Role or Contribution, eg, chair, principal investigator	Group (if more than 1 Group listed in the byline) and/or Subgroup (eg, Steering Committee)
Claudio	Moratti		MD	Neuroradiology Unit, Azienda Unità Sanitaria Locale - IRCCS di Reggio Emilia, Italy			TRISP
Manuela	Napoli		MD	Neuroradiology Unit, Azienda Unità Sanitaria Locale - IRCCS di Reggio Emilia, Italy			TRISP
Sheetal	Sumaria		MD	Stroke unit, University college hospitals NHS Foundation Trust			TRISP
Klaus	Gröschel		MD	Department of Neurology, University Medical Center of the Johannes Gutenberg University Mainz, Germany			TRISP
Marianne	Hahn		MD	Department of Neurology, University Medical Center of the Johannes Gutenberg University Mainz, Germany			TRISP
Alexandros	Tsp}qivmw		MD	Department of Neurology and Stroke Center, University Hospital Basel, Switzerland			TRISP
Christopher	Traenka		MD	HitevxqirxsjRiyvspsk}erh Girxiv0$Yrmzivwmx}$Lswtmxep$Fewip0$ W{mx~ivperh			TRISP
Annaelle	Zietz		MD	HitevxqirxsjRiyvspsk}erh Girxiv0$Yrmzivwmx}$Lswtmxep$Fewip0$ W{mx~ivperh			TRISP
Philippe	Lyrer		MD	HitevxqirxsjRiyvspsk}erh Girxiv0$Yrmzivwmx}$Lswtmxep$Fewip0$ W{mx~ivperh			TRISP
Gian Marco	DeMarchis		MD	HitevxqirxsjRiyvspsk}erh Girxiv0$Yrmzivwmx}$Lswtmxep$Fewip0$ W{mx~ivperh			TRISP
Sebastian	Thilemann		MD	HitevxqirxsjRiyvspsk}erh Girxiv0$Yrmzivwmx}$Lswtmxep$Fewip0$ W{mx~ivperh			TRISP

Supplemental Online Content: Nonauthor Collaborators
*First name, last name, and suffix (if applicable) are required and will appear in PubMed.

*First Name and Middle Initial(s)	*Last Name	*Suffix (eg, Jr, III)	Academic Degrees	Institution	Location (city, state/province, country)	Role or Contribution, eg, chair, principal investigator	Group (if more than 1 Group listed in the byline) and/or Subgroup (eg, Steering Committee)
Ines	Piot		MD	HitevxqirxsjRiyvspsk}erh Girxiv0$Yrmzivwmx}$Lswtmxep$Fewip0$ W{mx~ivperh			TRISP
Lukas	Enz		MD	HitevxqirxsjRiyvspsk}erh Girxiv0$Yrmzivwmx}$Lswtmxep$Fewip0$ W{mx~ivperh			TRISP
Nils	Peters		MD	HitevxqirxsjRiyvspsk}erh Girxiv0$Yrmzivwmx}$Lswtmxep$Fewip0$ W{mx~ivperh			TRISP
Mira	Katan		MD	HitevxqirxsjRiyvspsk}erh Girxiv0$Yrmzivwmx}$Lswtmxep$Fewip0$ W{mx~ivperh			TRISP
Alexander	Brehm		MD	HmekrswxmgerhMrxivzi Riyvsvehmspsk}0$Wxvsoi$Girxiv0$ Yrmzivwmx}$Lswtmxep$Fewip0$ W{mx~ivperh			TRISP
Marios	Psychogios		MD	HmekrswxmgerhMrxivzi Riyvsvehmspsk}0$Wxvsoi$Girxiv0$ Yrmzivwmx}$Lswtmxep$Fewip0$ W{mx~ivperh			TRISP
Ulrike	Prange		MD	Department of Neurology, Inselspital, Bern University Hospital, and University of Bern, Switzerland.			DOAC-IVT
Neil	Spratt		MD	Department of Neurology, John Hunter Hospital, NSW, Australia			TRISP
Laurent	Roten		MD	Department of Cardiology, Inselspital, Bern University Hospital, and University of Bern, Switzerland.			DOAC-IVT
BL Alvin	Chew		MD	Department of Neurology, John Hunter Hospital, NSW, Australia			DOAC-IVT

Supplemental Online Content: Nonauthor Collaborators

*First name, last name, and suffix (if applicable) are required and will appear in PubMed.

***First Name and Middle Initial(s)**	***Last Name**	***Suffix (eg, Jr, III)**	Academic Degrees	Institution	Location (city, state/province, country)	Role or Contribution, eg, chair, principal investigator	Group (if more than 1 Group listed in the byline) and/or Subgroup (eg, Steering Committee)
Jakub	Stefela		MD	Department of Neurology, Inselspital, Bern University Hospital, and University of Bern, Switzerland.			DOAC-IVT
Regina	von Rennenberg		MD	Department of Neurology with Experimental Neurology, Charité-Universitätsmedizin Berlin and Center for Stroke Research Berlin (CSB), Berlin, Germany			TRISP
Simon	Litmeier		MD	Department of Neurology with Experimental Neurology, Charité-Universitätsmedizin Berlin and Center for Stroke Research Berlin (CSB), Berlin, Germany			TRISP
Christoph	Riegler		MD	Department of Neurology with Experimental Neurology, Charité-Universitätsmedizin Berlin and Center for Stroke Research Berlin (CSB), Berlin, Germany			TRISP
Markus	Klammer		MD	Department of Neurology with Experimental Neurology, Charité-Universitätsmedizin Berlin and Center for Stroke Research Berlin (CSB), Berlin, Germany			TRISP
Hebun	Erdur		MD	Department of Neurology with Experimental Neurology, Charité-Universitätsmedizin Berlin and Center for Stroke Research Berlin (CSB), Berlin, Germany			TRISP
Hellwig	Simon		MD	Department of Neurology with Experimental Neurology, Charité-Universitätsmedizin Berlin and Center for Stroke Research Berlin (CSB), Berlin, Germany			TRISP

Supplemental Online Content: Nonauthor Collaborators
*First name, last name, and suffix (if applicable) are required and will appear in PubMed.

***First Name and Middle Initial(s)**	***Last Name**	***Suffix (eg, Jr, III)**	Academic Degrees	Institution	Location (city, state/province, country)	Role or Contribution, eg, chair, principal investigator	Group (if more than 1 Group listed in the byline) and/or Subgroup (eg, Steering Committee)
Magoni	Mauro		MD	Department of Neurological Sciences and Vision, ASST Spedali Civili, Brescia			TRISP
Longoni	Marco		MD	Department of Neuroscience, Neurology and Stroke Unit			TRISP
Cordici	Francesco		MD	Department of Neuroscience, Neurology and Stroke Unit			TRISP
Biguzzi	Sara		MD	Department of Neuroscience, Neurology and Stroke Unit			TRISP
Peter	Tan		MD	Department of Neuroscience, Eastern Health, VIC, Australia			TRISP
Marko	Ercegovac		MD	Neurology Clinic, Clinical Centre of Serbia, Faculty of Medicine, University of Belgrade, Belgrade, Serbia			TRISP
Ivana	Berisavac		MD	Neurology Clinic, Clinical Centre of Serbia, Faculty of Medicine, University of Belgrade, Belgrade, Serbia			TRISP
David	Haupenthal		MD	University Hospital Erlangen, Department of Neurology, Erlangen, Germany			TRISP
Luise	Gassmann		MD	University Hospital Erlangen, Department of Neurology, Erlangen, Germany			TRISP
Armin	Marsch		MD	University Hospital Erlangen, Department of Neurology, Erlangen, Germany			TRISP
Gabriela	Siedler		MD	University Hospital Erlangen, Department of Neurology, Erlangen, Germany			TRISP

*First name, last name, and suffix (if applicable) are required and will appear in PubMed.

***First Name and Middle Initial(s)**	***Last Name**	***Suffix (eg, Jr, III)**	Academic Degrees	Institution	Location (city, state/province, country)	Role or Contribution, eg, chair, principal investigator	Group (if more than 1 Group listed in the byline) and/or Subgroup (eg, Steering Committee)
Louise	Weir		MD	Department of Medicine and Neurology, Melbourne Brain Centre at the Royal Melbourne Hospital, University of Melbourne, Parkville, Victoria, Australia			DOAC-IVT
Lauren	Pesavento		MD	Department of Medicine and Neurology, Melbourne Brain Centre at the Royal Melbourne Hospital, University of Melbourne, Parkville, Victoria, Australia			DOAC-IVT
Nawaf	Yassi		MD	Department of Medicine and Neurology, Melbourne Brain Centre at the Royal Melbourne Hospital, University of Melbourne, Parkville, Victoria, Australia			DOAC-IVT
Stephen	Davis		MD	Department of Medicine and Neurology, Melbourne Brain Centre at the Royal Melbourne Hospital, University of Melbourne, Parkville, Victoria, Australia			DOAC-IVT
Bonaventure	Ip		MD	Department of Medicine and Therapeutics, Prince of Wales Hospital, The Chinese University of Hong Kong, Hong Kong.			DOAC-IVT
Thomas	Leung		MD	Department of Medicine and Therapeutics, Prince of Wales Hospital, The Chinese University of Hong Kong, Hong Kong.			DOAC-IVT
Alexandra	Gomez-Exposito		MD	Department of Neurology & Stroke. University of Tübingen, Tübingen, Germany			DOAC-IVT
Joshua	Mbroh		MD	Department of Neurology & Stroke. University of Tübingen, Tübingen, Germany			DOAC-IVT

*First name, last name, and suffix (if applicable) are required and will appear in PubMed.

***First Name and Middle Initial(s)**	***Last Name**	***Suffix (eg, Jr, III)**	Academic Degrees	Institution	Location (city, state/province, country)	Role or Contribution, eg, chair, principal investigator	Group (if more than 1 Group listed in the byline) and/or Subgroup (eg, Steering Committee)
Johannes	Tünnerhoff		MD	Department of Neurology & Stroke. University of Tübingen, Tübingen, Germany			DOAC-IVT
Gabriel	Barbu		MD	Department of Neurology & Stroke. University of Tübingen, Tübingen, Germany			DOAC-IVT
Yi	Wang		MD	Department of Neurology & Stroke. University of Tübingen, Tübingen, Germany			DOAC-IVT
Annerose	Mengel		MD	Department of Neurology & Stroke. University of Tübingen, Tübingen, Germany			DOAC-IVT
Feil	Katharina		MD	Department of Neurology & Stroke. University of Tübingen, Tübingen, Germany			DOAC-IVT
Markus	Kowarik		MD	Department of Neurology & Stroke. University of Tübingen, Tübingen, Germany			DOAC-IVT
Ulf	Ziemann		MD	Department of Neurology & Stroke. University of Tübingen, Tübingen, Germany			DOAC-IVT
Jun Yup	Kim		MD	Department of Neurology and Cerebrovascular Center, Seoul National University Bundang Hospital, Seoul National University College of Medicine, Seongnam, Republic of Korea			CRCS-K-NIH
Jihoon	Kang		MD	Department of Neurology and Cerebrovascular Center, Seoul National University Bundang Hospital, Seoul National University College of Medicine, Seongnam, Republic of Korea			CRCS-K-NIH

Supplemental Online Content: Nonauthor Collaborators
*First name, last name, and suffix (if applicable) are required and will appear in PubMed.

*First Name and Middle Initial(s)	*Last Name	*Suffix (eg, Jr, III)	Academic Degrees	Institution	Location (city, state/province, country)	Role or Contribution, eg, chair, principal investigator	Group (if more than 1 Group listed in the byline) and/or Subgroup (eg, Steering Committee)
Moon-Ku	Han		MD	Department of Neurology and Cerebrovascular Center, Seoul National University Bundang Hospital, Seoul National University College of Medicine, Seongnam, Republic of Korea			CRCS-K-NIH
Beom Joon	Kim		MD	Department of Neurology and Cerebrovascular Center, Seoul National University Bundang Hospital, Seoul National University College of Medicine, Seongnam, Republic of Korea			CRCS-K-NIH
Kang-Ho	Choi		MD	Department of Neurology, Chonnam National University Hospital, Gwangju, Republic of Korea			CRCS-K-NIH
Man-Seok	Park		MD	Department of Neurology, Chonnam National University Hospital, Gwangju, Republic of Korea			CRCS-K-NIH
Ki-Hyun	Cho		MD	Department of Neurology, Chonnam National University Hospital, Gwangju, Republic of Korea			CRCS-K-NIH
Joon-Tae	Kim		MD	Department of Neurology, Chonnam National University Hospital, Gwangju, Republic of Korea			CRCS-K-NIH
Dong-Ick	Shin		MD	Department of Neurology, Chungbuk National University Hospital, Cheongju, Republic of Korea			CRCS-K-NIH

Supplemental Online Content: Nonauthor Collaborators
*First name, last name, and suffix (if applicable) are required and will appear in PubMed.

***First Name and Middle Initial(s)**	***Last Name**	***Suffix (eg, Jr, III)**	Academic Degrees	Institution	Location (city, state/province, country)	Role or Contribution, eg, chair, principal investigator	Group (if more than 1 Group listed in the byline) and/or Subgroup (eg, Steering Committee)
Kyu Sun	Yum		MD	Department of Neurology, Chungbuk National University Hospital, Cheongju, Republic of Korea			CRCS-K-NIH
Dae-Hyun	Kim		MD	Department of Neurology, Dong-A University Hospital, Dong-A University College of Medicine, Busan, Republic of Korea			CRCS-K-NIH
Jae-Kwan	Cha		MD	Department of Neurology, Dong-A University Hospital, Dong-A University College of Medicine, Busan, Republic of Korea			CRCS-K-NIH
Jong-Moo	Park		MD	Department of Neurology, Uijeongbu Eulji Medical Center, Eulji University School of Medicine, Uijeongbu, Republic of Korea			CRCS-K-NIH
Kyusik	Kang		MD	Department of Neurology, Nowon Eulji Medical Center, Eulji University School of Medicine, Seoul, Republic of Korea			CRCS-K-NIH
Yong Soo	Kim		MD	Department of Neurology, Nowon Eulji Medical Center, Eulji University School of Medicine, Seoul, Republic of Korea			CRCS-K-NIH
Jae Guk	Kim		MD	Department of Neurology, Daejeon Eulji Medical Center, Eulji University School of Medicine, Daejeon, Republic of Korea			CRCS-K-NIH
Soo Joo	Lee		MD	Department of Neurology, Daejeon Eulji Medical Center, Eulji University School of Medicine, Daejeon, Republic of Korea			CRCS-K-NIH

Supplemental Online Content: Nonauthor Collaborators
*First name, last name, and suffix (if applicable) are required and will appear in PubMed.

***First Name and Middle Initial(s)**	***Last Name**	***Suffix (eg, Jr, III)**	Academic Degrees	Institution	Location (city, state/province, country)	Role or Contribution, eg, chair, principal investigator	Group (if more than 1 Group listed in the byline) and/or Subgroup (eg, Steering Committee)
Mi-Sun	Oh		MD	Department of Neurology, Hallym University Sacred Heart Hospital, Anyang, Republic of Korea			CRCS-K-NIH
Byung-Chul	Lee		MD	Department of Neurology, Hallym University Sacred Heart Hospital, Anyang, Republic of Korea			CRCS-K-NIH
Minwoo	Lee		MD	Department of Neurology, Hallym University Sacred Heart Hospital, Anyang, Republic of Korea			CRCS-K-NIH
Kyung-Ho	Yu		MD	Department of Neurology, Hallym University Sacred Heart Hospital, Anyang, Republic of Korea			CRCS-K-NIH
Hong-Kyun	Park		MD	Department of Neurology, Inje University Ilsan Paik Hospital, Inje University College of Medicine, Goyang, Republic of Korea			CRCS-K-NIH
Keun-Sik	Hong		MD	Department of Neurology, Inje University Ilsan Paik Hospital, Inje University College of Medicine, Goyang, Republic of Korea			CRCS-K-NIH
Yong-Jin	Cho		MD	Department of Neurology, Inje University Ilsan Paik Hospital, Inje University College of Medicine, Goyang, Republic of Korea			CRCS-K-NIH
Jay Chol	Choi		MD	Department of Neurology, Jeju National University Hospital, Jeju National University School of Medicine, Jeju, Republic of Korea			CRCS-K-NIH
Joong-Goo	Kim		MD	Department of Neurology, Jeju National University Hospital, Jeju National University School of Medicine, Jeju, Republic of Korea			CRCS-K-NIH

Supplemental Online Content: Nonauthor Collaborators
*First name, last name, and suffix (if applicable) are required and will appear in PubMed.

***First Name and Middle Initial(s)**	***Last Name**	***Suffix (eg, Jr, III)**	Academic Degrees	Institution	Location (city, state/province, country)	Role or Contribution, eg, chair, principal investigator	Group (if more than 1 Group listed in the byline) and/or Subgroup (eg, Steering Committee)
Chul-Hoo	Kang		MD	Department of Neurology, Jeju National University Hospital, Jeju National University School of Medicine, Jeju, Republic of Korea			CRCS-K-NIH
Sung Il	Sohn		MD	Department of Neurology, Keimyung University Dongsan Medical Center, Daegu, Republic of Korea			CRCS-K-NIH
Jeong-Ho	Hong		MD	Department of Neurology, Keimyung University Dongsan Medical Center, Daegu, Republic of Korea			CRCS-K-NIH
Seong Hwa	Jang		MD	Department of Neurology, Keimyung University Dongsan Medical Center, Daegu, Republic of Korea			CRCS-K-NIH
Hyungjong	Park		MD	Department of Neurology, Keimyung University Dongsan Medical Center, Daegu, Republic of Korea			CRCS-K-NIH
Tai Hwan	Park		MD	Department of Neurology, Seoul Medical Center, Seoul, Republic of Korea			CRCS-K-NIH
Sang-Soon	Park		MD	Department of Neurology, Seoul Medical Center, Seoul, Republic of Korea			CRCS-K-NIH
Kyung Bok	Lee		MD	Department of Neurology, Soonchunhyang University Hospital, Seoul, Republic of Korea			CRCS-K-NIH
Jee-Hyun	Kwon		MD	Department of Neurology, Ulsan University Hospital, Ulsan University College of Medicine, Ulsan, Republic of Korea			CRCS-K-NIH

Supplemental Online Content: Nonauthor Collaborators
*First name, last name, and suffix (if applicable) are required and will appear in PubMed.

***First Name and Middle Initial(s)**	***Last Name**	***Suffix (eg, Jr, III)**	Academic Degrees	Institution	Location (city, state/province, country)	Role or Contribution, eg, chair, principal investigator	Group (if more than 1 Group listed in the byline) and/or Subgroup (eg, Steering Committee)
Wook-Joo	Kim		MD	Department of Neurology, Ulsan University Hospital, Ulsan University College of Medicine, Ulsan, Republic of Korea			CRCS-K-NIH
Jun	Lee		MD	Department of Neurology, Yeungnam University Hospital, Daegu, Republic of Korea			CRCS-K-NIH
Doo Hyuk	Kwon		MD	Department of Neurology, Yeungnam University Hospital, Daegu, Republic of Korea			CRCS-K-NIH
Dong-Seok	Gwak		MD	Department of Neurology, Dongguk University Ilsan Hospital, Goyang, Republic of Korea			CRCS-K-NIH
Dong-Eog	Kim		MD	Department of Neurology, Dongguk University Ilsan Hospital, Goyang, Republic of Korea			CRCS-K-NIH
Keon-Joo	Lee		MD	Department of Neurology, Korea University Guro Hospital, Seoul, Republic of Korea			CRCS-K-NIH
Sang-Hwa	Lee		MD	Department of Neurology, Hallym University-Chuncheon Sacred Heart Hospital, Chuncheon, Gangwon-Do, Republic of Korea.			CRCS-K-NIH
Chulho	Kim		MD	Department of Neurology, Hallym University-Chuncheon Sacred Heart Hospital, Chuncheon, Gangwon-Do, Republic of Korea.			CRCS-K-NIH
Hae-Bong	Jeong		MD	Department of Neurology, Chung-Ang University Hospital, Seoul, Republic of Korea			CRCS-K-NIH
Kwang Yeol	Park		MD	Department of Neurology, Chung-Ang University Hospital, Seoul, Republic of Korea			CRCS-K-NIH

Supplemental Online Content: Nonauthor Collaborators
*First name, last name, and suffix (if applicable) are required and will appear in PubMed.

***First Name and Middle Initial(s)**	***Last Name**	***Suffix (eg, Jr, III)**	Academic Degrees	Institution	Location (city, state/province, country)	Role or Contribution, eg, chair, principal investigator	Group (if more than 1 Group listed in the byline) and/or Subgroup (eg, Steering Committee)
Ji Sung	Lee		MD	Clinical Research Center, Asan Medical Center, Seoul, Republic of Korea			CRCS-K-NIH
Juneyoung	Lee		MD	Department of Biostatistics, Korea University College of Medicine, Seoul, Republic of Korea			CRCS-K-NIH
Nicolas	Martinez-Majander		MD	Neurology, University of Helsinki and Helsinki University Hospital, Helsinki, Finland			TRISP
Gerli	Sibolt		MD	Neurology, University of Helsinki and Helsinki University Hospital, Helsinki, Finland			TRISP
Marjaana	Tiainen		MD	Neurology, University of Helsinki and Helsinki University Hospital, Helsinki, Finland			TRISP
Mauro	Gentile		MD	IRCCS Istituto Delle Scienze Neurologiche Di Bologna, Department of Neurology and Stroke Center, Maggiore Hospital, Bologna, Italy			TRISP
Ludovica	Migliaccio		MD	IRCCS Istituto Delle Scienze Neurologiche Di Bologna, Department of Neurology and Stroke Center, Maggiore Hospital, Bologna, Italy			TRISP
Luigi	Simonetti		MD	IRCCS Istituto Delle Scienze Neurologiche Di Bologna, Department of Neuroradiology, Maggiore Hospital, Bologna, Italy			TRISP

Supplemental Online Content: Nonauthor Collaborators
*First name, last name, and suffix (if applicable) are required and will appear in PubMed.

***First Name and Middle Initial(s)**	***Last Name**	***Suffix (eg, Jr, III)**	Academic Degrees	Institution	Location (city, state/province, country)	Role or Contribution, eg, chair, principal investigator	Group (if more than 1 Group listed in the byline) and/or Subgroup (eg, Steering Committee)
Matteo	Paolucci		MD	IRCCS Istituto Delle Scienze Neurologiche Di Bologna, Department of Neurology and Stroke Center, Maggiore Hospital, Bologna, Italy			TRISP
Alan	Flores		MD	Stroke Unit. Neurology department. Hospital Universitari Joan XXIII of Tarragona. Spain			TRISP
Laia	Seró		MD	Stroke Unit. Neurology department. Hospital Universitari Joan XXIII of Tarragona. Spain			TRISP
Guillaume	Turc		MD	Neurology Department, GHU Paris Psychiatrie et Neurosciences, INSERM U1266			TRISP
Catherine	Oppenheim		MD	Neuroradiology Department, GHU Paris Psychiatrie et Neurosciences, INSERM U1266			TRISP
Olivier	Naggara		MD	Neuroradiology Department, GHU Paris Psychiatrie et Neurosciences, INSERM U1266			TRISP
Wagih	Ben Hassen		MD	Neuroradiology Department, GHU Paris Psychiatrie et Neurosciences, INSERM U1266			TRISP
Joseph	Benzakoun		MD	Neuroradiology Department, GHU Paris Psychiatrie et Neurosciences, INSERM U1266			TRISP
Yusuke	Yakushiji		MD	Department of Neurology, Kansai Medical University Faculty of Medicine, Hirakata, Japan			DOAC-IVT
Takenobu	Kunieda		MD	Department of Neurology, Kansai Medical University Faculty of Medicine, Hirakata, Japan			DOAC-IVT
