# Supplementary material for: Intravenous Thrombolysis in Patients With Ischemic Stroke and Recent Ingestion of Direct Oral Anticoagulants
Source: JAMA Neurol. 2023 Jan 3;80(3):233–43. doi: 10.1001/jamaneurol.2022.4782 (PMC9857462; doi:10.1001/jamaneurol.2022.4782)
Supplement: Supplement 3. — Data Sharing Statement [file jamaneurol-e224782-s003.pdf]

Data Sharing Statement

Meinel. Intravenous Thrombolysis in Patients With Ischemic Stroke and Recent Ingestion of Direct Oral Anticoagulants. *JAMA Neurol*. Published January 03, 2023.
doi:10.1001/jamaneurol.2022.4782

Data
Data available: Yes
Data types: Deidentified participant data
How to access data: Data from this trial are currently not publicly available. We will share anonymized data upon reasonable request from any qualified investigator after clearance by the local ethics committee. A request for access to the data can be made by sending an email together with a research plan to david.seiffge@insel.ch .
When available: With publication

Supporting Documents
Document types: None

Additional Information
Who can access the data: qualified investigator after clearance by the local ethics committee
Types of analyses: any purpose
Mechanisms of data availability: without investigator support and after approval of a proposal and a signed data access agreement
Any additional restrictions: Swiss law, law of participating centers and regulations of respective ethics committees.
